# Thermally Activated Electric-Field Relay for Ultrafast and Stable NO_2_ Detection over Wide Temperature Range

**DOI:** 10.34133/research.1138

**Published:** 2026-02-16

**Authors:** Yucheng Ou, Bing Wang, Nana Xu, Quzhi Song, Tao Liu, Hui Xu, Fuwen Wang, Ming Zhang, Yingde Wang, Lei Liao

**Affiliations:** ^1^ Science and Technology on Advanced Ceramic Fiber and Composites Laboratory, College of Aerospace Science and Engineering, National University of Defense Technology, Changsha 410073, China.; ^2^ Changsha Semiconductor Technology and Application Innovation Research Institute, College of Semiconductors (College of Integrated Circuits), Hunan University, Changsha, China.

## Abstract

Conventional metal oxide sensors often suffer from limited long-term stability over an ultrabroad temperature range, primarily due to their single-type active sites and a static electronic configuration. To overcome this limitation, we constructed a dual local electric field (LEF) with a graded electron concentration profile by precisely modulating the local chemical environment of CeO_2_. This design introduces a thermally activated electric-field switching mechanism, which enables ultrafast and stable response value toward NO_2_ detection from −50 to 800 °C. We demonstrate that at low temperatures, the divergent hybridization between Pt and Ce orbitals leads to a lower thermal activation energy for LEF-1 than for LEF-2. As temperature rises, electron migration from the 4f orbitals of Ce^3+^ to adjacent Ce^4+^ weakens LEF-1, whereas thermal activation promotes efficient electron transfer in LEF-2, allowing LEF-2 to dominate at high temperatures and ensuring continuous activity. This relay sensing mechanism sustains rapid response (within 12 s) and long-term stability (over 75 d) at both −50 and 800 °C. This work presents an adaptive sensing mechanism through electron gradient differentiation and thermal-driven field switching, offering a new paradigm for the design of intelligent sensors under extreme conditions.

## Introduction

Metal oxide (MO) gas sensors are widely used in smart environmental monitoring, industrial internet of things, and aerospace systems [1–4]. However, their performance is fundamentally limited to single-type active sites and a static electronic structure. This limitation leads to sluggish reaction kinetics, irreversible surface passivation, sintering, phase transformation, and competitive adsorption when exposed to an ultrabroad temperature range [5–8]. Consequently, developing a sensor that delivers high sensitivity, ultrafast response, and exceptional long-term stability across such a wide thermal range remains a formidable challenge in materials science.

Previous research indicates that conventional approaches such as electronic compensation and dynamic thermal modulation exhibit inherent limitations including model hysteresis, high energy consumption, and slow response [9–11]. Breaking this performance ceiling requires a paradigm shift from static to dynamic sensing mechanisms. Inspired by biological systems that adapt to environmental changes, the next generation of intelligent sensors must feature self-regulated active sites capable of a functional “hand-off” across temperatures [12–14]. The performance of MO gas sensors is governed by the intricate interplay between surface chemical reactions and electronic properties [15–18]. Achieving temperature-adaptive detection in these sensors can be realized through dynamic modulation of local microstructures, enabling synchronous migration of electron excitation centers and gas adsorption sites [19–22]. Previous studies have demonstrated that the introduction of single atoms can induce localized reconstruction of the surface structure of MOs. Specifically, single-atom catalysts utilize inherent defect sites on the oxide support to anchor highly active individual atoms onto the oxide surface, thereby creating localized electron-rich regions on the MO surface [23–26]. However, randomly dispersed point defects are prone to migration under high-temperature conditions, which can lead to the aggregation of single atoms and result in instability of the localized electron-rich regions on the MO surface [27–29]. The strategic creation of ordered vacancy clusters offers a promising pathway [30]. The introduction of vacancy clusters effectively suppress defect migration, stabilize unusual coordination environments, and generate spatially organized electron density distributions, providing precise anchoring sites for single atoms [31–33]. Such an ordered defect structure underpins the establishment of active sites with divergent thermal activation energies, ultimately enabling a seamless “relay” of sensing function across temperatures without performance degradation.

In this study, we address this challenge by designing a model system comprising Pt single atoms (Pt_SAs_) anchored on vacancy clusters in CeO_2_ (v-CeO_2_). The novelty of this work lies in the creation of a dynamic sensing interface through a synergistic combination of ordered vacancy clusters and single atoms, which establishes a dual local electric field (LEF) system with a graded electron concentration profile. This unique configuration enables a thermally activated electric-field switching mechanism, an adaptive “relay” between LEF-1 that dominates at low temperatures and LEF-2 that dominates at high temperatures. In situ spectroscopy and theoretical calculations reveal a mechanism that enables continuous and efficient NO_2_ sensing. This yields unprecedented sensor performance: an ultrabroad operating temperature range (−50 to 800 °C) rapid response under 12 s and remarkable long-term stability over 75 d at both temperature extremes far surpassing conventional static sensors.

## Results and Discussion

### Structural characteristics

**Fig. 1.**
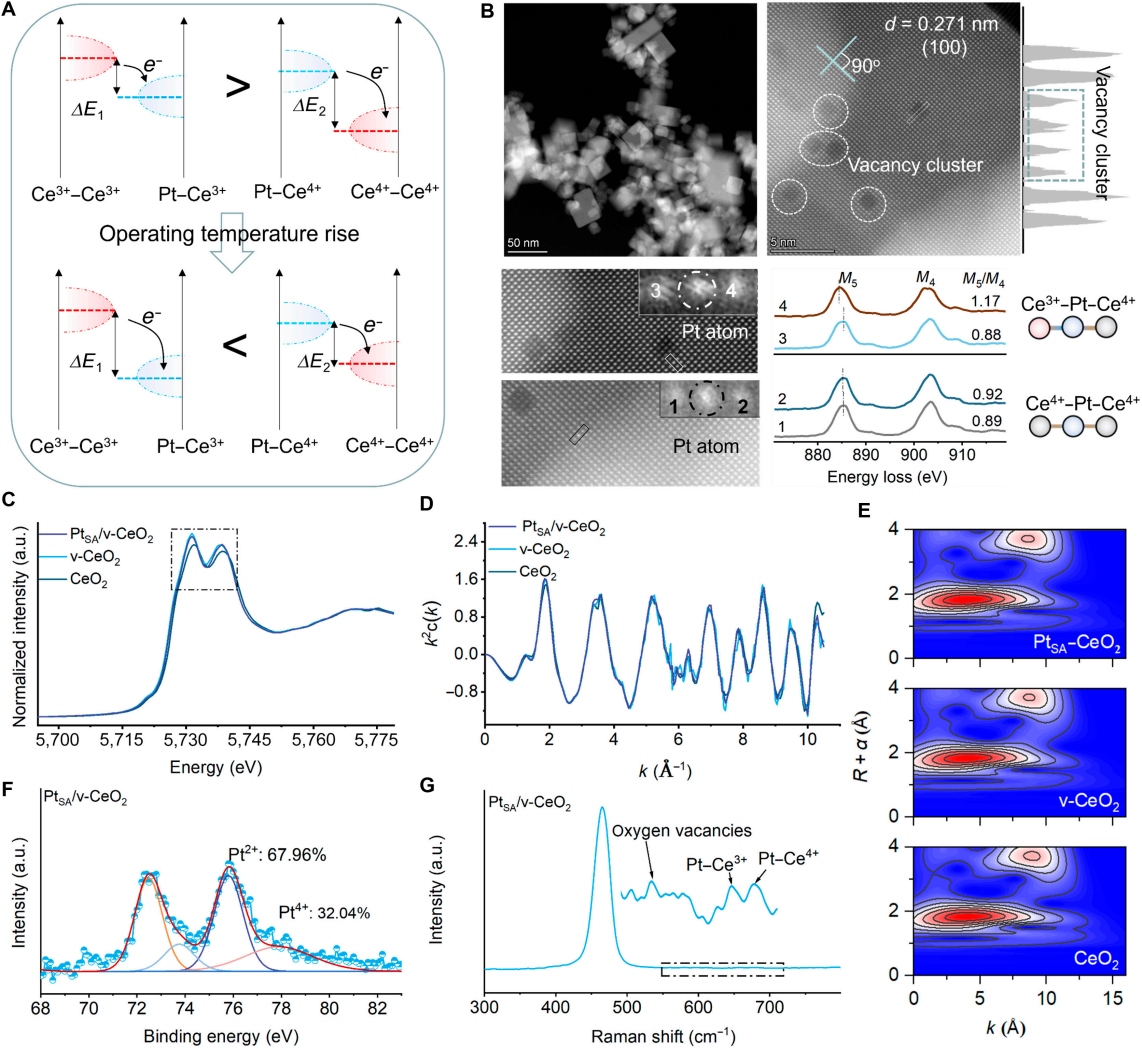
Structural characterization of Pt_SA_/v-CeO_2_. (A) Schematic diagram of temperature-activated electric-field switching. (B) AC-TEM and EELS analysis of Pt_SA_/v-CeO_2_. (C) Ce *L*_3_-edge XANES spectra of CeO_2_, v-CeO_2_, and Pt_SA_/v-CeO_2_. (D) Experimental and fitting results of Fourier transformed EXAFS spectra. (E) Wavelet transform from EXAFS signals at Ce *L*_3_-edge. (F) Pt 4f high-resolution XPS spectra. (G) Raman analysis of Pt_SA_/v-CeO_2_.

From the perspective of energy minimization and defect diffusion dynamics, the presence of ion vacancies on the (100)CeO_2_ surface reduces the coordination number of Ce atoms, which show higher mobility and increased surface energy. Moreover, the atomic ordering on the (100)CeO_2_ surface causes low-coordination Ce atoms and ion vacancies to migrate and form regular vacancy clusters at the atomic scale under high-temperature annealing [34]. This process creates more stepped edges and anchoring sites for Pt_SA_ (Fig. S1). X-ray diffraction (XRD) patterns confirm that the cubic phase structure remains intact after annealing treatments in both vacuum and air atmospheres (Fig. S2). Pt with a loading of 0.3 wt % is deposited on the surface of cubic-phase CeO_2_ (Fig. S3 and Table S1). In addition, the introduction of Pt_SA_ onto v-CeO_2_ inhibits the decrease in specific surface area caused by high-temperature treatment and enhances the structural stability of v-CeO_2_ (Figs. S4 and S5). The aberration-corrected transmission electron microscopy (AC-TEM) and atomic-resolution monochromated electron energy loss spectrum (EELS) reveal an important lower Ce^3+^ concentration in the edge regions of v-CeO_2_ compared to pristine CeO_2_ (Figs. S6 to S8). AC-TEM imaging of Pt_SA_/v-CeO_2_ confirmed the formation of ordered vacancy clusters from thermally activated single-ion vacancies in CeO_2_. It further revealed the preferential distribution of Pt_SA_ at the edges of these clusters and within defect-free crystalline regions (Fig. 1B and Fig. S9). The atomic-resolution monochromated EELS confirms that the elemental state of Ce atoms in the center regions of vacancy clusters is predominantly Ce^3+^ and the edge regions of vacancy clusters is predominantly Ce^4+^, which confirms that Pt_SA_ forms Pt_SA_–Ce^3+^ at the edge of vacancy clusters and Pt_SA_–Ce^4+^ at the perfect surface site, respectively. The x-ray absorption near-edge structure (XANES) and extended x-ray absorption fine structure (EXAFS) measurements confirm that the introduction of Pt_SA_ on surface of CeO_2_ results in a slightly decreased valence of Ce compared with v-CeO_2_, which confirms that the Pt_SA_ induced electron redistribution on CeO_2_ surface (Fig. 1C). The EXAFS fitting curves of the CeO_2_, v-CeO_2_ and Pt_SA_/v-CeO_2_ at *K* space confirm the presence of Ce–O and Ce–Ce bonding (Fig. 1D and Fig. S10). The wavelet transform pattern from EXAFS fitting curves confirms the introduction of Pt_SA_ on the surface of CeO_2_ causing the formation of Ce–O–Pt (S11). X-ray photoelectron spectroscopy (XPS) spectra confirm that the concentration of Ce^3+^ and oxygen concentration of CeO_2_ are similar to those in v-CeO_2_ and Pt_SA_/v-CeO_2_. The Pt_SA_ in Pt_SA_/v-CeO_2_ exists primarily as Pt^2+^ with a minor fraction of Pt^4+^, indicating 2 distinct coordination environments on the v-CeO_2_ surface (Fig. 1F and Fig. S12). Raman spectra of Pt_SA_/v-CeO_2_ confirm the presence of oxygen vacancies, Pt–Ce^3+^ and Pt–Ce^4+^ (Fig. 1G and Fig. S13) [35].

**Fig. 2.**
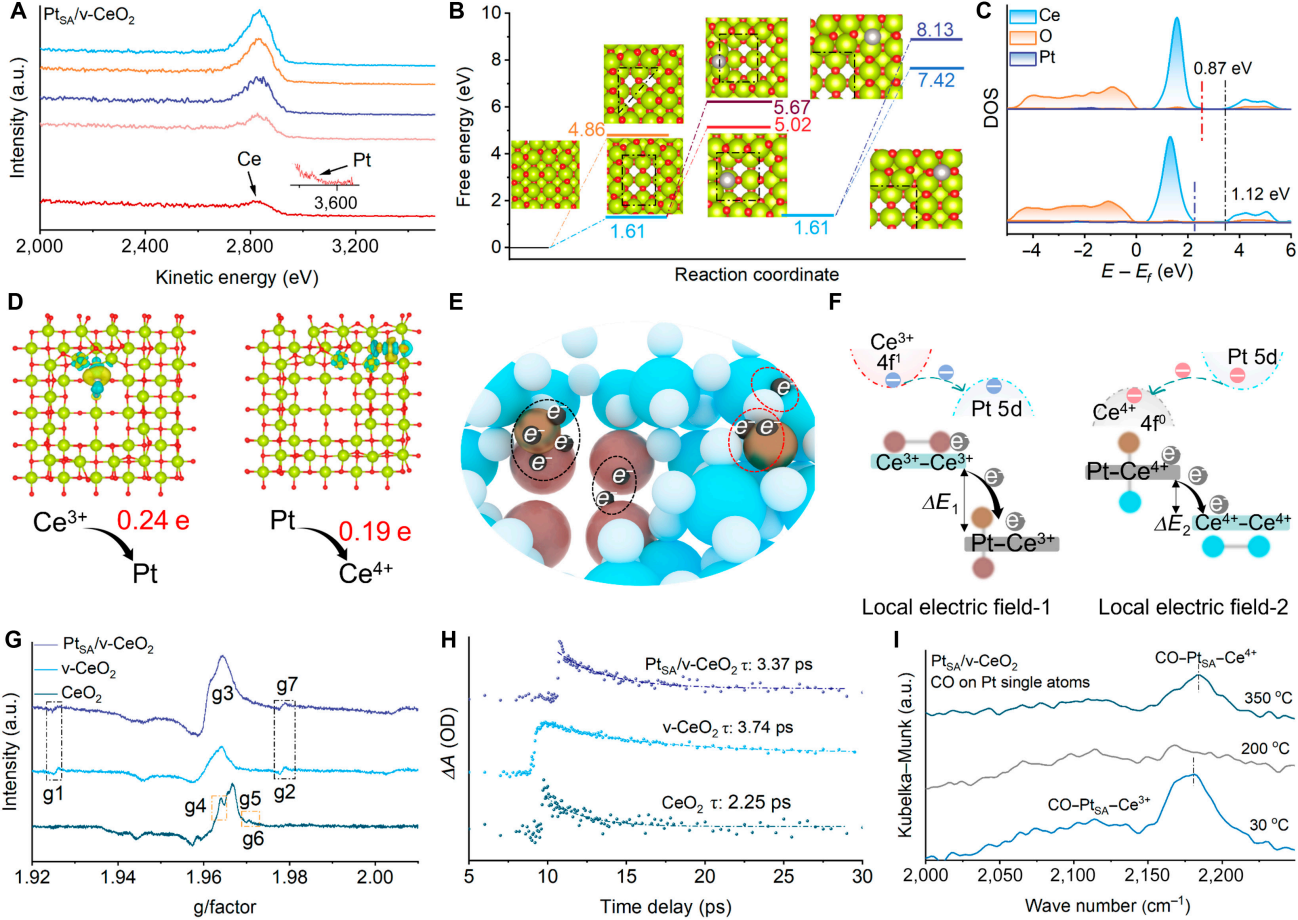
The formation mechanism of dual electric fields. (A) HS-LEIS analysis of Pt_SA_/v-CeO_2_. (B) Thermodynamics of the formation of dual electric fields. (C and D) DOS analysis and Bader charge difference distributions of Pt–Ce^3+^ and Pt–Ce^4+^. (E-F) Electron transfer mechanism diagram. (G) EPR analysis of CeO_2_, v-CeO_2_, and Pt_SA_/v-CeO_2_. (H) MIR-TAS of CeO_2_, v-CeO_2_, and Pt_SA_/v-CeO_2_. (I) In situ DRIFTS spectra of CO adsorption on Pt_SA_/v-CeO_2_. OD, optical density.

High-sensitivity, low-energy ion scattering (HS-LEIS) results show an important depletion of Ce atoms on the surface of Pt_SA_/v-CeO_2_ and indicate that Pt_SA_ is in contact with the outermost atomic layer (Fig. 2A). The exact structure of the Pt_SA_ is further explored via density functional theory (DFT), which show that the vacancy cluster is more inclined to square shape. The energy barrier for Pt_SA_ to occupy a 4-fold hollow site at the edge of a vacancy cluster is lower than that for adsorption on the edge, indicating that Pt_SA_ is more inclined to incorporate into defect sites, forming Pt_SA_–Ce^3+^ at the steps. Similarly, the Pt_SA_ is also occupying 4-fold hollow sites on the perfect surface site to form Pt_SA_–Ce^4+^ (Fig. 2B). The projected density of states (PDOS) of Pt_SA_–Ce^4+^ and Pt_SA_–Ce^3+^ confirms that Pt_SA_–Ce^3+^ exerts a markedly greater influence on the electronic structure near the Fermi level compared to Pt_SA_–Ce^4+^, with a higher electron concentration observed at Pt_SA_–Ce^3+^ sites (Fig. 2C). The Bader charge transfer between Pt_SA_ and CeO_2_ further confirms that the electron transfer between Pt_SA_ and Ce^3+^ is greater than that between Pt_SA_ and Ce^4+^ (Fig. 2D and Fig. S14). Then, the formation mechanism of LEF-1 and LEF-2 is shown in Fig. 2E. The construction of vacancy clusters promotes the generation of Ce^3+^–Ce^3+^ pairs, leading to strongly overlapping electron densities at the center of the vacancy cluster. This accelerates electron transfer from the bulk phase to the Pt_SA_–Ce^3+^ interface, resulting in the formation of LEF-1. Similarly, since the 4f orbitals of Ce^4+^ are unoccupied, while the 5d orbitals of Pt possess high electron density, electron transfer occurs from the Pt_SA_–Ce^4+^ to the Ce^4+^–Ce^4+^, resulting in the formation of LEF-2. The differences in electron concentration and transfer mechanisms between LEF-1 and LEF-2 result in a stronger LEF intensity for LEF-1 and a lower thermal activation energy for reactions occurring at its sites, compared to those of LEF-2.

Electron paramagnetic resonance (EPR) analysis confirms that the transformation of single-point defects into vacancy clusters promotes the conversion of high-coordination, low-activity Ce^3+^ on the CeO_2_ surface into low-coordination, high-activity Ce^3+^. In addition, the peak intensities of g1, g4, and g7 of Pt_SA_/v-CeO_2_ are stronger than those of v-CeO_2_, indicating that the formation of LEF-1 enhances the concentration of free electron in Ce^3+^ (Fig. 2F). The excited-state dynamics of charges in CeO_2_, v-CeO_2_, and Pt_SA_/v-CeO_2_ are explored via mid-infrared transient absorption spectroscopy (MIR-TAS). The relaxation time of free carriers varies in CeO_2_, v-CeO_2_, and Pt_SA_/v-CeO_2_ due to the presence of trapped states. The lifetimes of multiexponential fitting results confirm that the formation of LEF-1 and LEF-2 substantially enhances both the lifetime and transfer efficiency of carriers (Fig. 2G). Similarly, diffuse reflectance infrared Fourier transform spectroscopy (DRIFTS) using CO as a probe molecule confirmed that the difference in LEF strength results in a lower energy requirement for molecular adsorption at Pt_SA_–Ce^3+^ sites compared to Pt_SA_–Ce^4+^ sites (Fig. 2I).

### Gas performance

**Fig. 3.**
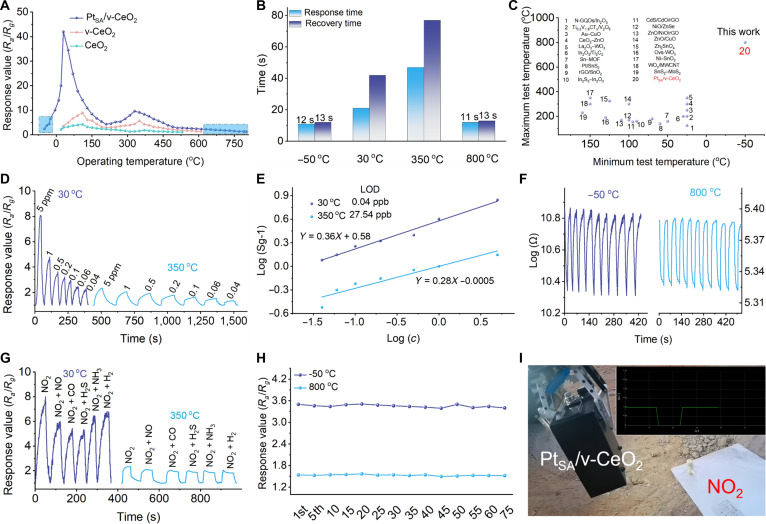
Gas sensing performance of Pt_SA_/v-CeO_2_. (A) Response value of CeO_2_, v-CeO_2_, and Pt_SA_/v-CeO_2_ toward NO_2_ detection under different operating temperatures. (B) Response–recovery time. (C) Reported operating temperature range of gas sensors in the literature. (D) Response values of Pt_SA_/v-CeO_2_ toward low-concentration NO_2_. (E) Response–concentration correlation. (F) Ten-cycle dynamic sensing response of Pt_SA_/v-CeO_2_ at −50 and 800 °C. (G) Selectivity performance. (H) Long-term stability of Pt_SA_/v-CeO_2_ at −50 and 800 °C. (I) Sensor device diagram.

To further evaluate the practical applicability of Pt_SA_/v-CeO_2_ under extreme operational conditions, we systematically investigated its work temperature range, response–recovery time, dynamic response characteristics, gas selectivity, and long-term stability performance. The sensing layers is authenticated to be 50 μm thick (Fig. S15). Figure 3A systematically compares the gas sensing performance of CeO_2_, v-CeO_2_ and Pt_SA_/v-CeO_2_ for NO_2_ detection across different operating temperatures. The response values show 2 peaks, indicating that the surface reaction mechanism of the sensitive layer changes correspondingly during the process of reaction chamber warming. CeO_2_ demonstrated a functional temperature range of 20 to 530 °C with dual optimal working temperatures at 110 and 370 °C. The introduction of vacancy clusters in v-CeO_2_ extended the operational range to 20 to 610 °C and substantially enhanced the response values across all temperatures. Most remarkably, subsequent Pt_SA_ functionalization on v-CeO_2_ further broadened the temperature range expansion to −50 to 800 °C and lowered the optimal working temperatures to 30 and 330 °C. In addition, the response values of Pt_SA_/v-CeO_2_ are higher than those of v-CeO_2_. The introduction of the dual LEF, combined with the catalytic effect of Pt_SA_, substantially enhances the concentration of surface free electrons and the adsorption and activation capabilities for NO_2_ and O_2_. This effectively reduces the reaction energy barrier, thereby lowering the optimal operating temperature. Figure 3B shows that the response–recovery times of Pt_SA_/v-CeO_2_ at −50 and 800 °C are 12 to 13 s and 11 to 13 s, respectively. Pt_SA_/v-CeO_2_ demonstrates superior performance metrics compared to previously reported gas sensors, exhibiting both an exceptionally broad operational temperature range and substantially shorter response–recovery time under extreme temperature conditions (Fig. 3C). Thus, simultaneously achieving fast and stable NO_2_ detection in wide temperature range has remained a challenging goal. The dynamic sensing response of Pt_SA_/v-CeO_2_ toward various concentrations of NO_2_ at 30 and 350 °C confirms that Pt_SA_–Ce^3+^, Ce^3+^–Ce^3+^, and Pt_SA_–Ce^4+^ exhibit good stability. The response values of Pt_SA_/v-CeO_2_ toward 40-part per billion (ppb) NO_2_ at 30 and 350 °C are 2.32 and 1.45, respectively. This performance indicates that the sensor is capable of detecting below the Environmental Protection Agency limit (53 ppb) (Fig. 3D). In addition, the Pt_SA_/v-CeO_2_ exhibits a positive linear response to NO_2_ in the range of 40 ppb to 5 parts per million. On the basis of the root mean square deviation method, the theoretical limit of detection of Pt_SA_/v-CeO_2_ is determined to be 0.04 and 27.54 ppb at 30 and 350 °C, respectively (Fig. 3E).

To evaluate the practical deployment of Pt_SA_/v-CeO_2_, we conduct 4 consecutive tests to measure its response values during both ascending temperatures from −50 to 800 °C and descending temperatures back to −50 °C. These assessments consistently highlighted Pt_SA_/v-CeO_2_ outstanding stability of response values and response–recovery time (Figs. S16 and S17). After 2 testing cycles, the CeO_2_ and Pt_SA_/CeO_2_ exhibited an important decrease in response values and prolonged response–recovery time, whereas the v-CeO_2_ maintained stable gas sensing performance throughout the cycling tests. This result clearly demonstrates that the formation of vacancy clusters enhances the stability of both surface Ce^3+^ and oxygen vacancies (Figs. S18 to S20). Pt_SA_/v-CeO_2_ also demonstrates excellent cyclic stability, with Pt_SA_/v-CeO_2_ showing a nearly fluctuation-free baseline resistance when repeatedly tested for 10 cycles at −50 and 800 °C (Fig. 3F). The introduction of Pt_SA_ induces reconstruction of the (100)CeO_2_ support surface, forming specific 4-fold hollow sites. The Pt_SA_ is firmly anchored to the support through the formation of robust Pt–O–Ce bonds. Simultaneously, the charge compensation effect resulting from the incorporation of Pt_SA_ into the (100)CeO_2_ lattice spontaneously generates and stabilizes the surrounding oxygen vacancies and Ce^3+^. Together, these factors constitute a thermodynamically stable composite active center, thereby enabling it to withstand harsh oxidative conditions. The XPS spectra and XRD pattern of Pt_SA_/v-CeO_2_ after reaction confirm the stability of Ce^3+^ (Fig. S21). Pt_SA_/v-CeO_2_ also demonstrates excellent cyclic stability. The gas sensing performance of Pt_SA_/v-CeO_2_ under different relative humidities is shown in Fig. S22. In the low-temperature region, the response value decreases markedly as the relative humidity increases. This is mainly attributed to the physical adsorption of water molecules on the CeO_2_ surface, which competes with NO_2_ for active sites and severely interferes with the gas sensing process. In contrast, in the high-temperature region, the sensing performance is insensitive to changes in humidity. This is because adsorbed water molecules are completely desorbed at high temperatures, greatly suppressing the competitive adsorption effect. Figure 3G shows the response values of Pt_SA_/v-CeO_2_ to interfering gases such as NO, CO, H_2_S, NH_3_, and H_2_ at the optimal operating temperature. While cross-sensitivity to interfering gases exists across the operational temperature range, their interfering influence is largely suppressed at the optimal sensing temperature. Here, the low activity of LEF-2 sites minimizes their direct response, whereas the NO_2_ maintains an important signal. This results in the high-selectivity pattern shown. The slightly diminished NO_2_ response compared to its value in pure gas is attributed to the inherent competitive adsorption and surface redox reactions common to MO-semiconductor-based sensing in mixed atmospheres. Therefore, operating at the LEF-2 stage effectively enhances selectivity by suppressing interferent signals. In addition, the long-term dynamic sensing response monitoring over 75 d of NO_2_ detection at −50 and 800 °C is shown Fig. 3H. It is found that the response value of Pt_SA_/v-CeO_2_ has hardly changed compared to the first day, indicating excellent long-term stability of the Pt_SA_/v-CeO_2_ at harsh temperature. To demonstrate the potential of Pt_SA_/v-CeO_2_ in extremely complex environments, we prepared a Pt_SA_/v-CeO_2_ sensor and transmitted the signal to the mobile phone via Bluetooth. The rover appears bumpy in the process of grabbing the sensor and moving, while the resistance of the sensor always remains stable, and there is no noise signal. When the sensor approaches, the sensor generates an electrical signal and transmits to the phone (Fig. 3I).

### Reaction mechanism

**Fig. 4.**
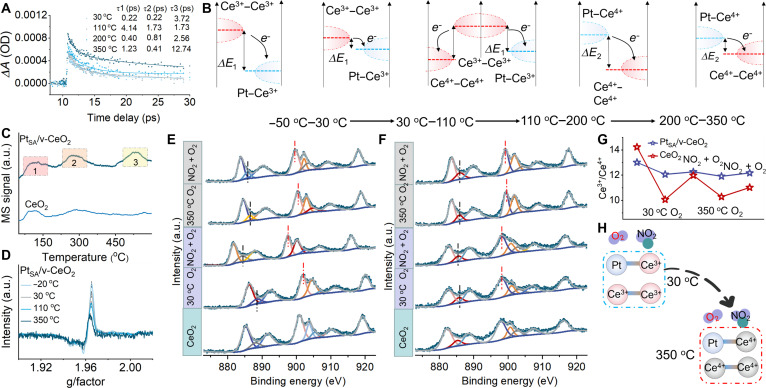
Reaction mechanism. (A) Temperature-dependent MIR-TAS of Pt_SA_/v-CeO_2_. (B) Schematic diagram of temperature-determined dual electric field relay mechanism. (C) Temperature-programmed desorption analysis. MS, mass spectrometry. (D) In situ EPR analysis of Pt_SA_/v-CeO_2_ under different temperatures. (E and F) In situ NAP-XPS analysis of CeO_2_ and Pt_SA_/v-CeO_2_ at different temperatures under O_2_ and NO_2_. (G) Proportional distribution of Ce valence states in each treatment from the peak-differentiating analysis of NAP-XPS results. (H) Surface adsorption schematic illustration of O_2_ and NO_2_ at Pt_SA_/v-CeO_2_ under different temperatures.

The LEF transfer mechanism with the increase in operating temperature is explored via MIR-TAS. The relaxation time of free carriers varies in different LEFs at different temperatures due to the presence of trapped states (Figs. S23 and S24). The lifetimes of multiexponential fitting results confirm that the excited-state absorbed signal has 3 attenuation times of Pt_SA_/v-CeO_2_, which correspond to the electron trap, interface electron transfer, and recombination process [36]. The attenuation times for Pt_SA_/v-CeO_2_ at 30 °C are 0.22 ps (τ1), 0.22 ps (τ2), and 3.72 ps (τ3), indicating that electron transfer at the interface dominates the dynamics and Pt_SA_ rapidly captures electrons from the 4f orbitals of Ce^3+^. With the temperature increased to 110 °C, the decay lifetimes are measured to be 4.14 ps (τ1), 1.73 ps (τ2), and 1.73 ps (τ3). The elevated temperature provides sufficient energy for electrons localized in the Ce^3+^ 4f orbitals to overcome the energy barrier associated with lattice relaxation, thereby facilitating their transfer from Ce^3+^ sites to adjacent Ce^4+^ sites. This redistribution leads to a reduction in electron concentration at Ce^3+^–Ce^3+^, resulting in a weakened electric field intensity at the LEF-1 interface and consequently a decrease in electron concentration and transfer efficiency. As the temperature further rises to 200 °C, the lifetimes become 0.40 ps (τ1), 0.81 ps (τ2), and 2.56 ps (τ3). The shortened τ1 and τ2 suggest a shift of the electron transfer center from LEF-1 to LEF-2. With the temperature increased to 350 °C, the attenuation times are 1.23 ps (τ1), 0.41 ps (τ2), and 12.74 ps (τ3), demonstrating that electron transfer at the LEF-2 interface again dominates the dynamics, with Ce^4+^ rapidly capturing electrons from Pt_SA_ (Fig. 4A and B). The temperature-programmed desorption analysis confirms that Pt_SA_/v-CeO_2_ has more reaction sites and stronger gas molecule capacity compared with CeO_2_ (Fig. 4C). In situ EPR analysis shows that the increase in temperature causes the conversion of Ce^3+^ to Ce^4+^, while the formation of vacancy cluster improves the stability of Ce^3+^ in v-CeO_2_ compared with CeO_2_ (Fig. S25). In addition, the peak position of g3 in Pt_SA_/v-CeO_2_ is shifted with the increase in temperature, further confirming the strong interaction between Pt_SA_ and Ce^3+^ (Fig. 4D).

The dynamic transformation process of the reaction site in CeO_2_ and Pt_SA_/v-CeO_2_ is explored via in situ near ambient pressure (NAP)-XPS (Fig. 4E and F). The reaction of electrons with O_2_ in CeO_2_ to produce active oxygen causes the peaks of Ce^3+^ and Ce^4+^ shift to higher binding energy. Then, the peaks of Ce^3+^ and Ce^4+^ in CeO_2_ shift to lower binding energy when NO_2_ enters the reaction chamber, indicating that Ce^3+^-Ce^4+^ redox couples dominate the adsorption and activation of target molecules at 30 °C. With the operating temperature increased to 350 °C, the peak positions of Ce^3+^ and Ce^4+^ also shift to lower binding energies when NO_2_ enters the reaction chamber, confirming that Ce^3+^–Ce^4+^ redox couples continue to play a role in O_2_ and NO_2_ adsorption. Concurrently, the concentration of oxygen vacancy is decrease from 15.65% to 5.67% (Fig. S26). The formation of active oxygen also shifts the Ce^3+^ peak to higher binding energies in Pt_SA_/v-CeO_2_. Then, the peaks of Ce^3+^ in Pt_SA_/v-CeO_2_ shift to lower binding energy when NO_2_ enters the reaction chamber, suggesting that the LEF-1 plays the main role in adsorption–activation target molecules at 30 °C. On the contrary, with the operating temperature increased to 350 °C, the peak position of Ce^3+^ remains unchanged, while the peaks of Ce^4+^ in Pt_SA_/v-CeO_2_ shift to lower binding energies when NO_2_ enters the reaction chamber, indicating a transition to LEF-2 as the primary active site. As shown in Fig. 4G, the variation in peak area ratios of Ce^3+^/Ce^4+^ on the surfaces of CeO_2_ and Pt_SA_/v-CeO_2_ exhibits a clear dependence on both temperature and reaction atmosphere. The generation of active oxygen causes the peak area ratio of Ce^3+^/Ce^4+^ in CeO_2_ decreased from 14.23% to 10.06%. Then, the peak area ratio of Ce^3+^/Ce^4+^ increased from 10.06% to 11.98% when NO_2_ enters the reaction chamber. With the operating temperature increased to 350 °C, the peak area ratio of Ce^3+^/Ce^4+^ in CeO_2_ decreased from 11.98% to 10.28% under O_2_ atmosphere, with NO_2_ raising it to 11.02%. In contrast, the introduction of vacancy clusters and Pt_SA_ substantially enhances the stability of Ce^3+^. The peak area ratio of Ce^3+^/Ce^4+^ in Pt_SA_/v-CeO_2_ only decreases from 13.01% to 12.06% under an O_2_ atmosphere, and NO_2_ marginally increases it to 12.26%. With the operating temperature increased to 350 °C, O_2_ lowers the ratio to 11.91%, while NO_2_ restores it to 12.17%. Hence, the surface adsorption schematic illustration of O_2_ and NO_2_ in Pt_SA_/v-CeO_2_ at different operating temperatures is shown in Fig. 4H. The LEF-1 is the main adsorption sites toward NO_2_ detection in the low-temperature region. With the increase in temperature, the adsorption site is transferred from LEF-1 to LEF-2. The change of baseline resistance of all samples at different operating temperatures further confirms the phenomenon of LEF migration (Fig. S27). The baseline resistance detected by Pt_SA_/v-CeO_2_ for NO_2_ can be restored to the original state at 30 °C, while the baseline resistance detected by Pt_SA_/v-CeO_2_ for NO_2_ cannot be restored to the original state at 200 °C. During the electric field transition, the decrease in surface reactivity prevents the conversion of NO_2_ into stable products and leads to secondary reactions during the recovery process, which causes to baseline resistance drift.

### Theoretical results

**Fig. 5.**
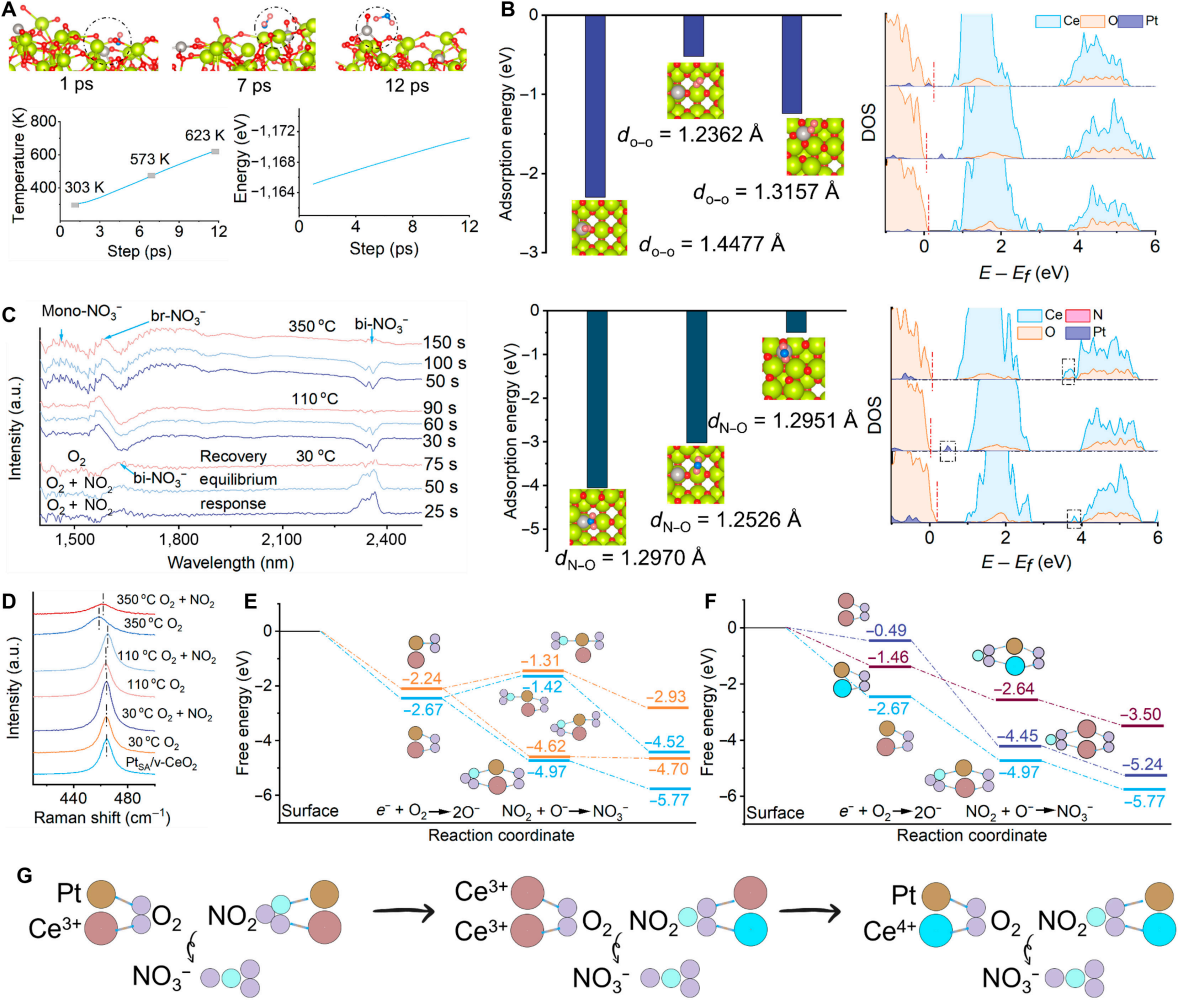
Theoretical calculation and analysis. (A) MD analysis. (B) Adsorption energy and DOS. (C) In situ FTIR spectra. (D) In situ Raman spectra. (E and F) Reaction energy (*E*_a_) and reaction model for the reaction of NO_2_ with active oxygen. (G) Reaction mechanism diagram.

The molecular dynamics (MD) analysis also confirms that the reaction center is transferred from LEF-1 to LEF-2 with the increase in operating temperature (Fig. 5A). According to the MD analysis, the adsorption–activation methods of LEF-1 and LEF-2 toward O_2_ and NO_2_ are explored via DFT (Figs. S28 and S29). The O_2_ adsorption method in LEF-1 can be divided into Pt_SA_–O–O–Ce^3+^, side-on Pt_SA_–O_2,_ end-on Pt_SA_–O_2_, and Ce^3+^–O–O–Ce^3+^. The adsorption energy and the length of the O–O bond confirm that O_2_ is more inclined to Pt–O–O–Ce^3+^ adsorption mode. The O_2_ adsorption method in LEF-2 can be divided into Pt_SA_–O–O–Ce^4+^ and end-on Pt_SA_–O_2_. The adsorption energy and change in the length of the O–O bond indicate that O_2_ is more inclined to Pt_SA_–O–O–Ce^4+^ adsorption mode (Fig. 5B). In addition, PDOS of O_2_ adsorption in Pt_SA_/v-CeO_2_ at LEF-1 confirms that the formation of Pt_SA_–Ce^3+^ in CeO_2_ causes the presence of Pt 5d electron states at the Fermi level, which promotes charge transfer from Ce^3+^ to Pt_SA_ and further reaction with O_2_ to produce active oxygen. The formation of Pt_SA_–Ce^4+^ also enhances the concentration of electrons and reduces the energy required to excite electrons from the O 2p state to the Ce 5d state, while the charges are transferred from Pt_SA_ to Ce^4+^ and further react with O_2_ (Fig. S30). In addition, the generation of pit can increase the local electron density and promote the electron transfer between Ce^3+^ and O_2_. Similarly, the NO_2_ adsorption energy and change in the length of N–O bond in LEF-1 confirm that NO_2_ is more inclined to Pt_SA_–N–O–O–Ce^3+^ adsorption mode. The NO_2_ adsorption methods in LEF-2 are more inclined to O–Pt_SA_–O–N. The PDOS of NO_2_ adsorption on Pt_SA_/v-CeO_2_ also confirms the otherness of the electron transfer method between the NO_2_ and different reaction sites (Fig. S31). The in situ Fourier transform infrared (FTIR) spectroscopy is conducted to analyze NO_2_ reaction process on the surface of CeO_2_, v-CeO_2_, and Pt_SA_/v-CeO_2_ (Figs. S32 and S33). At 30 °C, the O_2_ can react with electrons to produce active oxygen, which subsequently reacts with NO_2_ to generation NO_3_^−^ during the reaction process. The peaks at 1,627 and 2,366 cm^–1^ are indicative of the formation of bidentate nitrate (bi-NO_3_^−^) [37,38]. During the reaction equilibrium process, the intensity of bi-NO_3_^−^ remains constant [39]. During the recovery process, the absorbance intensity of bi-NO_3_^−^ at 2,366 cm^–1^ substantially decreases because of the desorption of NO_2_.

With the operating temperature increased to 110 °C, the product on the Pt_SA_/v-CeO_2_ surface remained bi-NO_3_^−^, but the peak intensity is substantially lower compared to that at 30 °C. This suggests a reduction in the electric field strength of LEF-1, leading to decreased concentrations of surface free electrons and active oxygen species. As the temperature is further raised to 350 °C, a large amount of unstable monodentate nitrate (mono-NO_3_^−^) and bridge nitrate (br-NO_3_^−^), along with a small amount of bi-NO_3_^−^, is generated on the Pt_SA_/v-CeO_2_ surface. The in situ FTIR results demonstrate that the active sites shift from LEF-1 to LEF-2 with increasing temperature. Although the concentration of active oxygen generated by LEF-2 at high temperatures is lower than that generated by LEF-1 at low temperatures, it is sufficient to drive the sensing reaction at the elevated optimal temperature. Then, the surface reaction mechanism in Pt_SA_/v-CeO_2_ is deeply explored via in situ Raman (Fig. 5D). The peak at 464.49 cm^−1^ is typical F2g of Pt_SA_/v-CeO_2_. The blue shift of the F2g peak of Pt_SA_/v-CeO_2_ occurs in O_2_ atmosphere due to lattice shrinkage and oxygen vacancy consumption, resulting in 2 Ce^4+^ ions (0.97 Å) replacing 2 Ce^3+^ ions (ionic radius, 1.14 Å). The peaks at 264 and 596 cm^−1^ are linked with the oxygen vacancies, which also confirm that the variation trend of oxygen vacancy concentration is consistent with the shift of F2g. The peaks at 1,052 and 1,159 cm^−1^ are linked with the generation of O^−^ and O_2_^−^, confirming the presence of surface reaction between O_2_ and electrons [40]. Then, the introduction of NO_2_ into the reaction chamber caused a red shift in the F2g peak, which confirmed that the reaction of NO_2_ with O_2_ can produce electrons leading to the reduction of Ce^4+^ to Ce^3+^. In addition, the peak intensity of oxygen vacancies also increases with the introduction of NO_2_. The peak position of F2g exhibits a blue shift with the operating temperature increased to 50 °C under O_2_ atmosphere, and the peak intensity of oxygen vacancies decreases. Then, the introduction of NO_2_ in the reaction chamber also caused the phenomenon of red shift in F2g peak, and the peak intensity of oxygen vacancies also increased. The peak intensities of oxygen vacancies and active oxygen in Pt_SA_/v-CeO_2_ substantially decrease when the operating temperature reaches 350 °C, indicating that the concentration and reaction activity of Ce^3+^ decrease. In particular, the introduction of NO_2_ in the reaction chamber at 350 °C does not increase the oxygen vacancy concentration, indicating that the reaction site in Pt_SA_/v-CeO_2_ has changed from LEF-1 to LEF-2. The generation of active oxygen in CeO_2_ surface also changes from O_2_^−^ to O^−^ with the operating temperature reaching higher values (Figs. S34 and S35). In addition, in situ Raman analysis confirms that the reaction sites of CeO_2_ are mainly concentrated at a single active site Ce^3+^ in the high-operating-temperature region.

Activation barriers at different reaction sites and different LEFs in Pt_SA_/v-CeO_2_ are further explored via DFT (Fig. 5E and F). First, the reaction process of O_2_ on pit center in Pt_SA_/v-CeO_2_ is parallel adsorption at Ce^3+^–Ce^3+^ site. Then, the adsorption O_2_ can react with electrons to spontaneously generate active oxygen on the surface of pit center, and the reaction energy is −0.49 eV. Then, the active oxygen can further react with NO_2_ to generate NO_3_^−^, and the reaction energy is −4.45 eV. Second, the reaction process of O_2_ on pit edge in Pt_SA_/v-CeO_2_ can be divided into the adsorption of O_2_ at the site of Pt_SA_–Ce^3+^ to form Pt_SA_–O–O–Ce^3+^ and the parallel adsorption of O_2_ on the surface of Pt_SA_. The reaction energies are −2.67 and −2.24 eV, respectively. Similarly, the adsorption methods of NO_2_ on pit edge in Pt_SA_/v-CeO_2_ can also be divided into the adsorption of NO_2_ at the site of Pt_SA_–Ce^3+^ to form Pt_SA_–N–O–O–Ce^3+^ and the parallel adsorption of NO_2_ on the surface of Pt_SA_. The reaction energies required for the reaction of Pt_SA_–O–O–Ce^3+^ with Pt_SA_–N–O–O–Ce^3+^ and N–O–Pt_SA_–O are −5.77 and −4.52 eV. The reaction energies required for the reaction of O–Pt_SA_–O with Pt_SA_–N–O–O–Ce^3+^ and N–O–Pt_SA_–O are −4.70 and −2.93 eV. Third, the reaction process of O_2_ in Pt_SA_/v-CeO_2_ at the site of Pt_SA_–Ce^4+^ forms Pt_SA_–O–O–Ce^4+^, with a reaction energy of −1.46 eV. Then, the active oxygen can react with NO_2_ to produce NO_3_^−^, with a reaction energy of −3.50 eV. Therefore, within the temperature range of −50 to 800 °C, the reaction modes of O_2_ and NO_2_ transition from Pt_SA_–O–O–Ce^3+^ and Pt_SA_–N–O–O–Ce^3+^ to Ce^3+^–O–O–Ce^3+^ and Ce^3+^–O–N–O–Ce^3+^ in LEF-1 and ultimately revert to Pt_SA_–O–O–Ce^4+^ and Pt_SA_–O–N–O–Ce^4+^ in LEF-2.

## Conclusion

In summary, we have precisely modulated the local chemical environment of CeO_2_ by constructing ordered vacancy clusters to anchor Pt_SA_, thereby establishing dual LEFs with a gradient electron concentration distribution. This design enables rapid and stable NO_2_ detection across an ultrawide temperature range from −50 to 800 °C. We demonstrate that distinct Pt–Ce orbital hybridization results in a lower thermal activation energy required for LEF-1 at low temperatures compared to LEF-2. Within the range of −50 to 110 °C, LEF-1 dominates, leveraging its high electron density and low activation energy barrier to facilitate superior molecular adsorption and reaction kinetics. As the temperature increases (110 to 200 °C), partial electron transfer occurs from Ce^3+^–Ce^3+^ pairs at the vacancy cluster centers to adjacent Ce^4+^ sites, weakening the Pt–Ce orbital hybridization effect within LEF-1 and reducing electron transfer efficiency. Nevertheless, the elevated temperature supplies sufficient thermal energy to activate LEF-2, accelerating interfacial electron transfer and enabling LEF-2 to dominate above 200 up to 800 °C. Leveraging this electric-field relay mechanism, Pt_SA_/v-CeO_2_ exhibits remarkable long-term stability over the entire temperature range from −50 to 800 °C and achieves NO_2_ detection within 12 s even at −50 and 800 °C. This work provides a new strategy for designing advanced sensing systems through atomic-scale manipulation of defect clusters and LEFs.

## Materials and Methods

### Synthesis of Pt_SA_/v-CeO_2_

v-CeO_2_ (0.1 g) was dispersed in 30 ml of distilled water under stirring. As the powder disperses evenly, 0.2 wt % of Pt solution (H_12_N_6_O_6_Pt) was added. Then, the mixed solution was stirred for 12 h, and the powder was collected and washed. Finally, the powder was heat-treated at 800 °C for 10 h under flowing dry air.

### Fabrication and testing of a gas sensor and experimental detail

Pt_SA_/v-CeO_2_ (5 mg) was mixed with 50 μl of deionized water to obtain the corresponding slurry. Slurry (5 μl) was then dripped onto Pt interdigitated electrodes (10 mm × 5 mm × 0.25 mm; Aurora Technologies, China) to form a resistance-type sensor. All the fabricated sensors were aged in air at 80 °C for 2 h. The gas sensing performance of the fabricated sensors was evaluated using an intelligent gas sensing analysis system (CGS-4TPs, Beijing Elite Technology Co. Ltd.). To produce test gases with the necessary concentrations, a dynamic gas and liquid distribution system (DGL-III, Beijing Elite Technology Co. Ltd., China) having 3 mass flow controllers was used. We used nitrogen as the carrier gas and controlled the gas flow of oxygen and nitrogen to achieve different oxygen concentrations in the reaction chamber. The interference gas was injected into the reaction chamber through a syringe.The room housing the gas-sensing analysis system was equipped with a constant temperature and humidity air-conditioning system, and the indoor humidity could be artificially controlled by setting parameters. The response value (*S*_g_) is defined as the ratio of the resistance in air (*R*_a_) to that in target gas (*R*_g_). The response time and recovery time are defined as the time for the sensor to reach 90% of the final signal.

### Characterization and measurements

XRD patterns were recorded on a Bruker D8 x-ray powder diffractometer with Cu Kα radiation (λ = 1.5418 Å) at 30 kV and 10 mA with a scanning rate of 5°·min^−1^ in the 2θ range of 10° to 80°. The high-resolution TEM images were taken on a Talos F200i working at 200 kV and JEOL JEM-2100F field-emission TEM with an accelerating voltage of 200 kV. The high-angle annular dark-field scanning TEM images and x-ray energy-dispersive spectroscopy (EDS) mapping were recorded on AC-TEM (FEI Titan Cubed Themis G2 300) at an accelerating voltage of 300 kV. An EDS detector with a solid angle of 0.13 sr and a Fiori number >4,000 was used. A spectral map of 460 pixels was acquired with a dwell time of 1.85 s per pixel, resulting in a total acquisition time of 851 s. The in situ FTIR spectra were recorded on a Bruker Vertex 70 FTIR spectrometer equipped with in situ reaction chamber. X-band EPR measurement was performed at room temperature using a JEOL FA-200 EPR spectrometer. The XPS (Axis Supra) measurements were operated with Al Ka radiation (1,486.6 eV). Binding energies were calibrated by setting the measured binding energy of C 1s to 284.8 eV. In situ DRIFTS spectra were measured on a Nicolet-6700 FTIR spectrometer. The N_2_ adsorption–desorption isotherms were measured using a BELSORP-max-II to estimate specific surface area and pore size distribution by the Brunauer–Emmett–Teller and Barrett–Joyner–Halenda methods. The process Raman system used in this study was HORIBA. XAFS measurements were measured on the B11 station in Shanghai Synchrotron Radiation Facility. HS-LEIS spectra were acquired at room temperature using a high-sensitivity analyzer (ION-TOF Qtac100). To minimize the damage to the surface, helium was selected as the ion source with a kinetic energy of 3 keV, an ion flux of 6,000 pA·m^−2^, and a spot size of 2 mm × 2 mm.

### X-ray absorption data analysis

The obtained XAFS data were processed in Athena (version 0.9.26) for background, preedge line and postedge line calibrations. Then, Fourier transformed fitting was carried out in Artemis (version 0.9.26). *k*^2^ weighting with a *k* range of 2 to 10 Å^−1^ and an *R* range of 1 to 3 Å was used for the fitting of CeO_2_ and the sample.

### Mid-infrared transient absorption spectroscopy

TAS measurements were performed using a femtosecond amplifier laser system (Spitfire Ace, Spectra-Physics), which generated 35-fs laser pulses centered at 800 nm with a repetition rate of 1 kHz. The output laser was split into 3 beams. The first beam was used to pump an optical parametric amplifier (TOPAS, Spectra-Physics) to generate pump pulses with tunable wavelengths from visible to MIR range. The second beam was used to generate MIR supercontinuum pulses as the MIR probe, by simultaneously focusing the 800-nm fundamental light and the second-harmonic light at 400 nm into air. The MIR probe was detected by a liquid-nitrogen-cooled 64-element mercury–cadmium–telluride-coupled spectrometer (FPAS-0144, Infrared Systems Development). The TAS measurements were conducted with a pump wavelength of 1.2 μm and a probe wavelength of 5 μm. Temperature change equipment is from the Linkam Scientific, Temperature Control Stage TS1500.

### DFT calculations

DFT calculations were carried out using the VASP code. The projector augmented-wave method and Perdew–Burke–Ernzerhof generalized gradient approximation were used for the exchange correlation functionals. The energy cutoff of 400 eV was used. The MD simulations were carried out in the canonical ensemble (NVT) with the Nose–Hoover thermostat to generate amorphous models. The time step was set to 1 fs. All initial amorphous structures were thermally equilibrated at ambient temperature for 10 ps. The energy and force on each ion were reduced below 10^−5^ eV·atom^−1^ and 0.01 eV·Å^−1^, respectively, and only the Γ point was sampled from the Brillouin zone. The supercell system consists of 96 O, 46 Ce, 2 Pt, and 1 N. The amorphous models were obtained using a heat-up process: from 324 to 774 K within 10 ps to obtain the structural snapshots and total energy distributions at the corresponding temperatures. The effect of core electrons on the density of valence electrons was described using the projector augmented-wave method. The kinetic energy cutoff for the plane waves was set to 450 eV for all the calculations. To consider the open-shell d electrons, generalized gradient approximation plus Hubbard U schemes were implemented, using effective *U* values of 5.0 for Ce. The convergence tolerances for energy and force on each atom during structure relaxation were less than 10^−5^ eV and 0.03 eV·A^−1^, respectively. A set of Monkhorst–Pack *K*-point meshes of 2 × 2 × 1 and 4 × 4 × 1 was used to sample the Brillouin zone for geometry optimization and electronic structure calculations. A vacuum distance of 15 Å was set to ensure sufficient vacuum and avoid interactions between 2 periods. Further calculations were carried out to determine the thermal and zero-point energy corrections at the Γ point of various intermediates adsorbed on the surface. The VASPKIT code was used for postprocessing computational data obtained from VASP.

The adsorption energy, *ΔG*_ads_, can be evaluated, which is defined as

$${\Delta E}_{\mathrm{ads}}=E_{\left(\text{System}+\mathrm{gas}\right)}-E_{\left(\text{System}\right)}-1/2E_{\mathrm{ads}},$$

in which *E*_(System+ gas)_ and *E*_(System)_ are the energies of all research systems with and without gas adsorption, respectively. *E*_ads_ represents the energy of adsorbed intermediates.

## Data Availability

Relevant data supporting the key findings of this study are available within the article and the Supplementary Materials. All raw data generated during the current study are available from the corresponding authors upon request.

## References

[1] Zhong YH, Yuan GT, Bao DQ, Tao Y, Gao ZQ, Zhao W, Li S, Yang YT, Zhang PP, Zhang H, et al. Specific Sn-O-Fe active sites from atomically Sn-doping porous Fe_2_O_3_ for ultrasensitive NO_2_ detection. Nano Micro Lett. 2025;17(1):276. 10.1007/s40820-025-01770-9 PMC12104129 40415065

[2] Tung TT, Nine MJ, Krebsz M, Pasinszki T, Coghlan CJ, Tran DNH, Losic D. Recent advances in sensing applications of graphene assemblies and their composites. Adv Funct Mater. 2017;27(46):1702891.

[3] Manohar A, Suvarna T, Vattikuti SVP, Sudhani HPK, Manivasagan P, Jang ES, Al-Asbahi BA, Mameda N, Kim KH. Ternary nanocomposites of ZnFe_2_O_4_/NiFe_2_O_4_/CeO_2_: Investigating electrochemical energy storage and biocompatible properties. J Environ Chem Eng. 2024;12(5): Article 113337.

[4] Manohar A, Suvarna T, Vattikuti SVP, Sudhani HPK, Manivasagan P, Jang ES, Shaikh SF, Kumar A, Sharma K, Mameda N, et al. Comprehensive study of CeO_2_/CuFe_2_O_4_ nanocomposites: Structural, EPR, magnetic, electrochemical, and cytotoxicity properties. Mater Charact. 2024;218(1): Article 114471.

[5] Liu YX, Parisi J, Sun XC, Lei Y. Solid-state gas sensors for high temperature applications-a review. J Mater Chem A. 2014;2(26):9919–9943.

[6] Lin H, Luo C, Cheng FY, Xie K. Engineering active interfaces on the surface of porous single-crystalline TiO_2_ monoliths for enhanced catalytic activity and stability. Research. 2025;8:0579. 39810854 10.34133/research.0579 PMC11729270

[7] Huang HL, Du ZT, Wu HC, Gao FM, Jiang L, Hou HL, Chen SL, Li WJ, Hu F, Yang WY, et al. High-temperature resistant ethanol sensing enhanced by ZnO nanoparticles/SiC nanowire heterojunctions. Appl Surf Sci. 2024;645: Article 158828.

[8] Xu JY, Fan XX, Xu KC, Wu KD, Liao HL, Zhang C. Ultrasensitive chemiresistive gas sensors based on dual-mesoporous zinc stannate composites for room temperature rice quality monitoring. Nano Micro Lett. 2025;17(1):115. 10.1007/s40820-024-01645-5 PMC11759721 39853638

[9] Zhou SX, Yao L, Zhao T, Mei H, Dassios KG, Cheng LF, Zhang LT. Chemiresistively sensitized SiOC structure for formaldehyde detection under thermal and pressure loading. Carbon. 2023;201:100–109.

[10] Zhou SX, Yao L, Mei H, Lu MY, Cheng LF, Zhang LT. Strengthening PPy/TiO_2_ arrayed SiOC honeycombs for self-protective gas sensing. Compos Part B Eng. 2022;230: Article 109536.

[11] Manohar A, Suvarna T, Vattikuti SVP, Kim D, Sangaraju S, Al-Asbahi BA, Kim KH. Zn_0.5_Mg_x_Cu_0.5-x_Fe_2_O_4_ spinel ferrites as electrode materials for supercapacitor applications. Inorg Chem Commun. 2015;177: Article 114393.

[12] Manohar A, Suvarna T, Vattikuti SVP, Manivasagan P, Jang ES, Sudhani HPK, Al-Enizi AM, Kumar A, Sharma K, Mameda N, et al. Structural, morphological, magnetic, electrochemical and biocompatible properties of ZnFe_2_O_4_/MgFe_2_O_4_/NiFe_2_O_4_/CeO_2_ nanocomposites. Colloids Surf, A. 2015;705(2): Article 135667.

[13] Ohodnicki PR, Buric MP, Brown TD, Matranga C, Wang CJ, Baltrus J, Andio M. Plasmonic nanocomposite thin film enabled fiber optic sensors for simultaneous gas and temperature sensing at extreme temperatures. Nanoscale. 2013;5(19):9030–9039. 23948985 10.1039/c3nr02891g

[14] Chu M, Wang X, Wang X, Lou X, Zhang C, Cao M, Wang L, Li Y, Liu S, Sham TK, et al. Site-selective polyolefin hydrogenolysis on atomic Ru for methanation suppression and liquid fuel production. Research. 2023;6:0032. 37040499 10.34133/research.0032 PMC10076030

[15] Yang MS, Bae SK, Seo DB, Lee K, Son Y, Kim SH, Jung JH, An KS, Park I, Seo JH. Facile formation of nanoporous reduced graphene oxide via epoxy-based negative photoresist laser irradiation for highly sensitive and selective gas detection. Sensor Actuat B Chem. 2025;426: Article 137073.

[16] Wang J, Yang H, Li F, Li LG, Wu JB, Liu SH, Cheng T, Xu Y, Shao Q, Huang XQ. Single-site Pt-doped RuO_2_ hollow nanospheres with interstitial C for high-performance acidic overall water splitting. Sci Adv. 2022;8(9): Article eabl9271. 35235348 10.1126/sciadv.abl9271 PMC8890715

[17] Manohar A, Suvarna T, Vattikuti SVP, Goud JP, Al-Asbahi BA, Qaid SMH, Mameda N, Kim KH. Investigating the potential of Mg_0.5_Zn_x_Cu_0.5-x_Fe_2_O_4_ nanoparticles for energy storage applications. Mat Sci Semicon Proc. 2025;185: Article 108897.

[18] Martín AJ, Mitchell S, Mondelli C, Jaydev S, Pérez-Ramírez J. Unifying views on catalyst deactivation. Nat Catal. 2022;5(10):854–866.

[19] Ren Q, He Y, Wang H, Sun YJ, Dong F. Rapid energy exchange between in situ formed bromine vacancies and CO_2_ molecules enhances CO_2_ photoreduction. Research. 2023;6:0244. 37808179 10.34133/research.0244 PMC10557117

[20] Qu DY, Liu TP, Cheng YY, Du T, Cheng BL, Zhang Y, Su C, Zheng YB, Xu X, Wang G, et al. Volatilomics in diseases odour and electronic nose diagnosis. Trac-Trend Anal Chem. 2025;193: Article 118440.

[21] Hejazi S, Mohajernia S, Osuagwu B, Zoppellaro G, Andryskova P, Tomanec O, Kment S, Zboril R, Schmuki P. On the controlled loading of single platinum atoms as a Co-catalyst on TiO_2_ anatase for optimized photocatalytic H_2_ generation. Adv Mater. 2020;32(16):1908505. 10.1002/adma.201908505 32125728

[22] Zhou QQ, Ding QH, Geng ZX, Hu CC, Yang L, Kan ZT, Dong B, Won M, Song HW, Xu L. A flexible smart healthcare platform conjugated with artificial epidermis assembled by three-dimensionally conductive MOF network for gas and pressure sensing. Nano Micro Lett. 2025;17(1):50. 10.1007/s40820-024-01548-5 PMC11511809 39453552

[23] Qu DY, Cheng BL, Shao XY, Hu JY, Bai S, Zhang Y, Wu WW, Haick H. Advances in metals and metal hybrids-based gas sensors and their applications. Rare Metals. 2025; 10.1007/s12598-025-03527-0

[24] Shi X, Dai C, Wang X, Hu J, Zhang J, Zheng L, Mao L, Zheng H, Zhu M. Protruding Pt single-sites on hexagonal ZnIn_2_S_4_ to accelerate photocatalytic hydrogen evolution. Nat Commun. 2022;13(1):1287. 35277495 10.1038/s41467-022-28995-1 PMC8917206

[25] Zhang RF, Xue B, Tao YH, Zhao HQ, Zhang ZX, Wang XN, Zhou XY, Jiang B, Yang ZL, Yan XY, et al. Edge-site engineering of defective Fe-N_4_ nanozymes with boosted catalase-like performance for retinal vasculopathies. Adv Mater. 2022;34(39):2205324. 10.1002/adma.202205324 35953446

[26] Xu J, Shao GL, Tang X, Lv F, Xiang HY, Jing CF, Liu S, Dai S, Li YG, Luo J, et al. Frenkel-defected monolayer MoS_2_ catalysts for efficient hydrogen evolution. Nat Commun. 2022;13(1):2193. 35459263 10.1038/s41467-022-29929-7 PMC9033855

[27] Li X, Pereira-Hernández XI, Chen YZ, Xu J, Zhao JK, Pao CW, Fang CY, Zeng J, Wang Y, Gates BC, et al. Functional CeO*_x_* nanoglues for robust atomically dispersed catalysts. Nature. 2022;611(7935):284. 36289341 10.1038/s41586-022-05251-6

[28] Li RT, Xu XY, Zhu BE, Li XY, Ning YX, Mu RT, Du PF, Li MW, Wang HK, Liang JJ, et al. In situ identification of the metallic state of Ag nanoclusters in oxidative dispersion. Nat Commun. 2021;12(1):1406. 33658489 10.1038/s41467-021-21552-2 PMC7930130

[29] Aitbekova A, Zhou CS, Stone ML, Lezama-Pacheco JS, Yang AC, Hoffman AS, Goodman ED, Huber P, Stebbins JF, Bustillo KC, et al. Templated encapsulation of platinum-based catalysts promotes high-temperature stability to 1100°C. Nat Mater. 2022;21(11):1290. 36280703 10.1038/s41563-022-01376-1

[30] Wu ZY, Zhu P, Cullen DA, Hu YF, Yan QQ, Shen SC, Chen FY, Yu HR, Shakouri M, Arregui-Mena JD, et al. A general synthesis of single atom catalysts with controllable atomic and mesoporous structures. Nat Synth. 2022;1(8):658–667.

[31] Lu F, Yi D, Liu SJ, Zhan F, Zhou B, Gu L, Golberg D, Wang X, Yao JN. Engineering platinum-oxygen dual catalytic sites via charge transfer towards highly efficient hydrogen evolution. Angew Chem Int Ed. 2020;59(40):17865–17871. 10.1002/anie.202008117 32621558

[32] Zhang YM, Zhao JH, Wang H, Xiao B, Zhang W, Zhao XB, Lv TP, Thangamuthu M, Zhang J, Guo Y, et al. Single-atom Cu anchored catalysts for photocatalytic renewable H_2_ production with a quantum efficiency of 56%. Nat Commun. 2022;13(1):2062. 35411001 10.1038/s41467-022-29799-z PMC9001637

[33] Yang BP, Liu K, Li HJW, Liu CX, Fu JW, Li HM, Huang JE, Ou PF, Alkayyali T, Cai C, et al. Accelerating CO_2_ electroreduction to multicarbon products via synergistic electric-thermal field on copper nanoneedles. J Am Chem Soc. 2022;144(7):3039–3049. 35112839 10.1021/jacs.1c11253

[34] Li XY, Wang C, Tang JW. Methane transformation by photocatalysis. Nat Rev Mater. 2022;7(8):617–632.

[35] Xie PF, Ding J, Yao ZH, Pu TC, Zhang P, Huang ZN, Wang CH, Zhang JL, Zecher-Freeman N, Zong H, et al. Oxo dicopper anchored on carbon nitride for selective oxidation of methane. Nat Commun. 2022;13(1):1375. 35296655 10.1038/s41467-022-28987-1 PMC8927601

[36] Ou YC, Wang B, Xu NN, Song QZ, Liu T, Xu H, Wang FW, Wang YD. Crystal face-dependent behavior of single-atom Pt: Construct of SA-FLP dual active sites for efficient NO_2_ detection. Adv Sci. 2024;11(29):2402038. 10.1002/advs.202402038 PMC11304280 38810152

[37] Wang M, Hong XW, Chen JJ, Li JH, Chen XP, Mi JX, Liu ZM, Xiong SC. Two-step hydrothermal synthesis of highly active MnO_x_-CeO_2_ for complete oxidation of formaldehyde. Chem Eng J. 2022;440: Article 135854.

[38] Feng YL, Tan XL, Shi ZJ, Villamena FA, Zweier JL, Song YG, Liu YP. Trityl quinodimethane derivatives as unimolecular triple-function extracellular EPR probes for redox, pH, and oxygen. Anal Chem. 2023;95(2):1057–1064. 36602544 10.1021/acs.analchem.2c03754

[39] Zhang XD, Yue K, Rao RZ, Chen JF, Liu Q, Yang Y, Bi FK, Wang YX, Xu JC, Liu N. Synthesis of acidic MIL-125 from plastic waste: Significant contribution of N orbital for efficient photocatalytic degradation of chlorobenzene and toluene. Appl Catal B Environ. 2022;310: Article 121300.

[40] Qin LB, Sun F, Gong ZH, Ma GY, Chen Y, Tang Q, Qiao L, Wang RH, Liu ZQ, Tang ZH. Electrochemical NO_3_^−^ reduction catalyzed by atomically precise Ag_30_Pd_4_ bimetallic nanocluster: Synergistic catalysis or tandem catalysis? ACS Nano. 2023;17(13):12747–12758. 37377221 10.1021/acsnano.3c03692

