# Supplementary material for: Thermally Activated Electric-Field Relay for Ultrafast and Stable NO2 Detection over Wide Temperature Range
Source: Research (Wash D C). 2026 Feb 16;9:1138. doi: 10.34133/research.1138 (PMC12907473; doi:10.34133/research.1138)
Supplement: Supplementary 1 — Figs. S1 to S35 Table S1 to S4 [file research.1138.f1.doc]

**Thermally activated electric-field relay for ultra-fast and stable NO_2_ detection over wide temperature range**

Yucheng Ou^a^, Bing Wang^a*^,Nana Xu^a^, Quzhi Song^a^, Tao Liu^a^, Hui Xu^a^, Fuwen Wang^a^, Ming Zhang^b^, Yingde Wang^a^, Lei Liao^b^

*^a^Science and Technology on Advanced Ceramic Fiber and Composites Laboratory, College of Aerospace Science and Engineering,* *National University of Defense Technology, Changsha 410073, China*

*^b^Changsha Semiconductor Technology and Application Innovation Research Institute, College of Semiconductors (College of Integrated Circuits), Hunan University, Changsha, China*

**Corresponding authors:*

*E-Mail: bingwang@nudt.edu.cn*

**Contents**

**I. Experimental Section**

**II. Supplementary Figures**

**Figs. S1 to S32**

**III. Supplementary Tables**

**Tables. S1 to S4**

**Experimental Section**

**Methods**

**Chemicals and Materials.**

Tetraammineplatinum(II) nitrate (H_12_N_6_O_6_Pt, 99.99%), and Cerous nitrate hexahydrate (Ce(NO_3_)_3_·6H_2_O, 99.95%) were purchased from Aladdin, sodium hydroxide (NaOH, ≧98%) were purchased from Aladdin. The water used in all experiments was ultrapure (18.2 Ω/cm).

**Synthesis of CeO_2_.** CeO_2_ was synthesized by hydrothermal method as well. 0.868 g Ce(NO_3_)_3_·6H_2_O and 9.6 g NaOH are dissolved in 35 mL and 5 ml of distilled water, respectively. As the clear solution formed, NaOH aqueous solution is slowly added to the Ce(NO_3_)_3_·6H_2_O with vigorous stirring. After stirring for about 30 mins, and mixture solution was subjected to hydrothermal treatment at 180 ^o^C for 24 h. The obtained powders were washed with water and ethanol for three cycles, dried in vacuum over a night, and further subjected to calcination at 550 ^o^C for 4 h.

**Synthesis of v-CeO_2_.** v-CeO_2_ are synthesized by the high temperature heat treatment method. 0.1 g CeO_2_ are heat treatment at 800^o^C for 1 h under vacuum.

**II. Supplementary Figure**


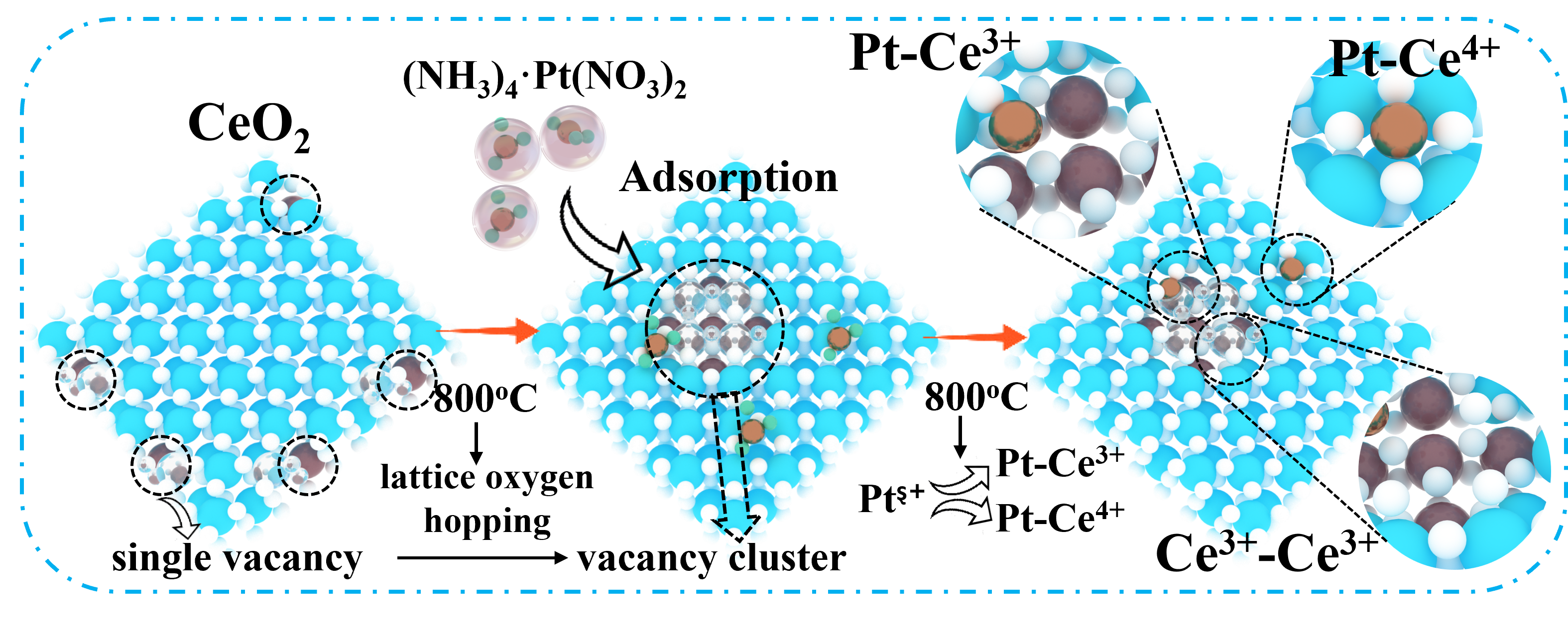


**Fig. S1** Schematic illustration of synthesis for Pt_SA_/v-CeO_2_.


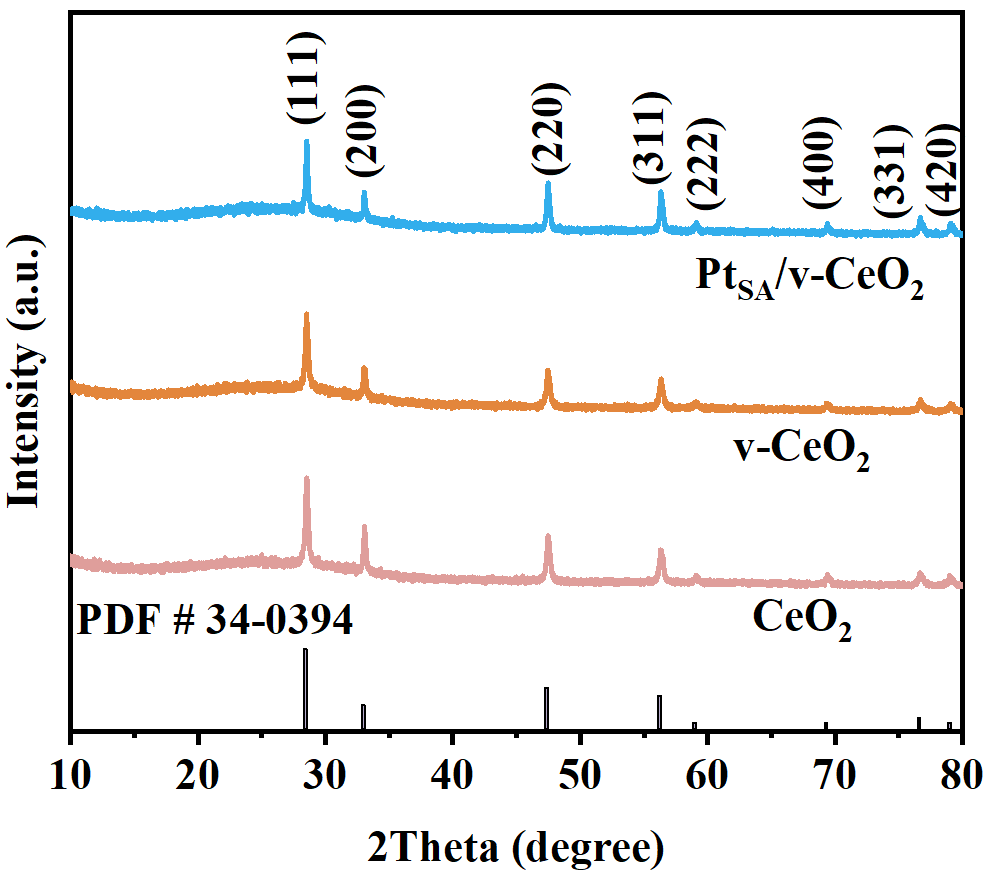


**Fig. S2** The XRD pattern of CeO_2_, v-CeO_2_ and Pt_SA_/v-CeO_2_.


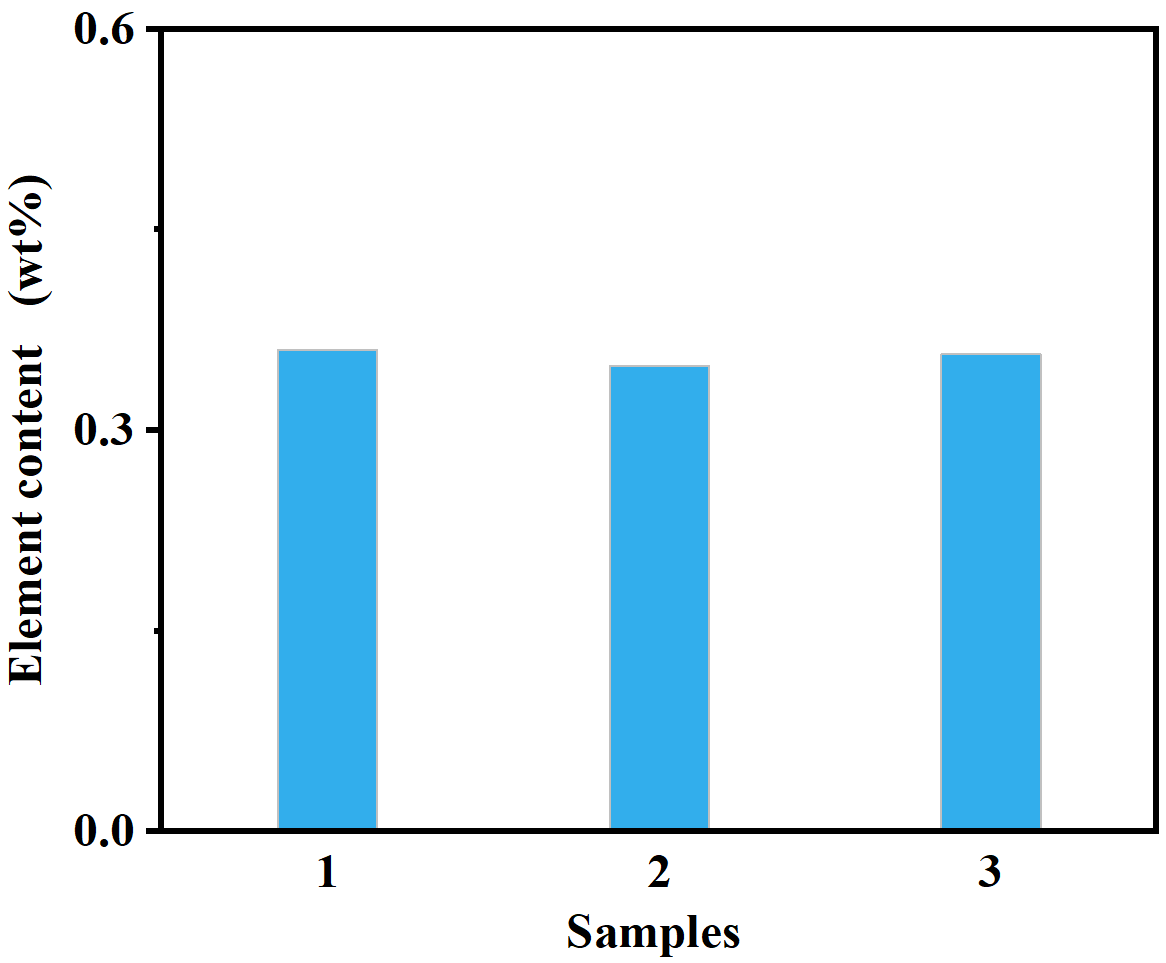


**Fig. S3** The element content of Pt in Pt_SA_/v-CeO_2_.


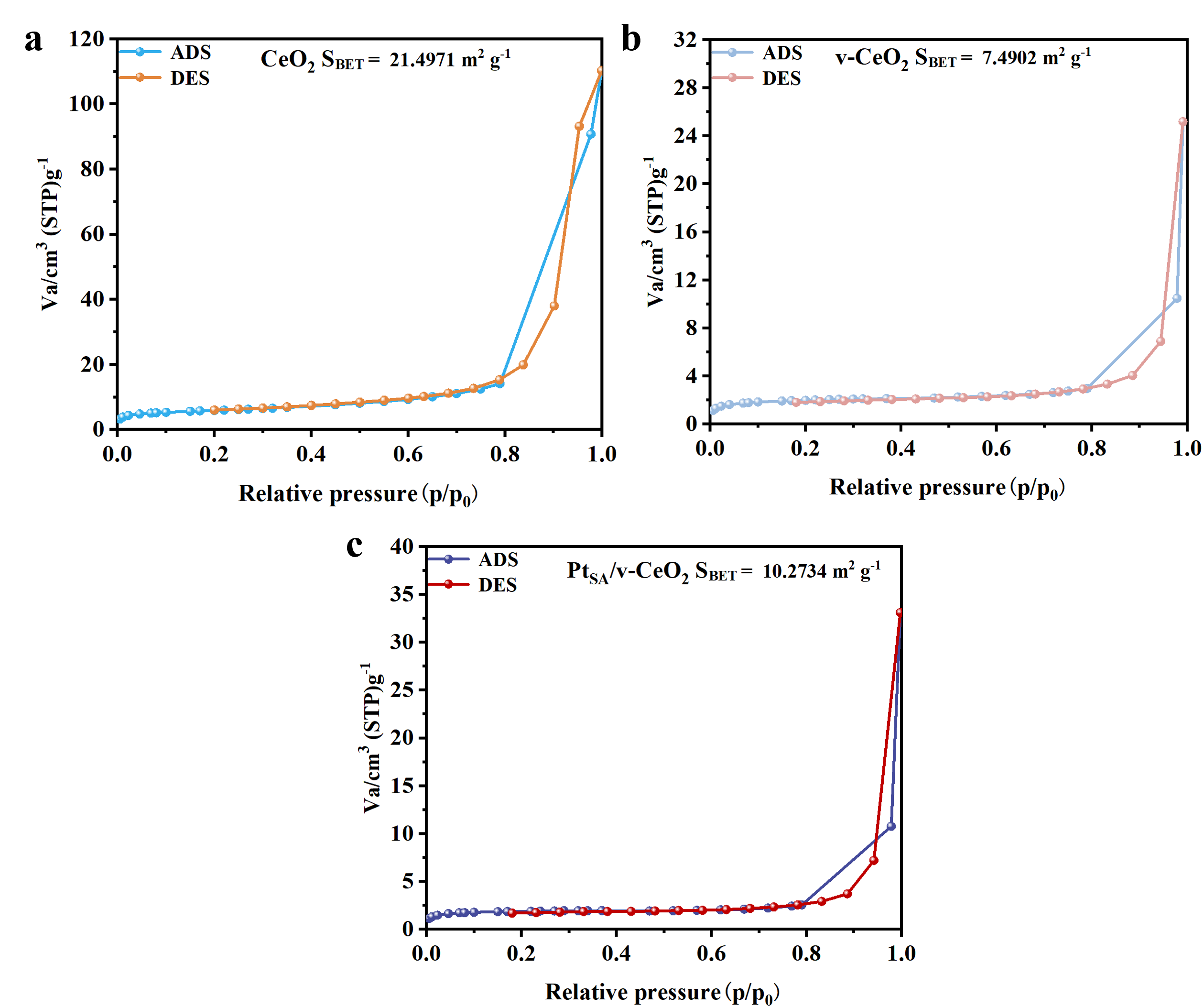


**Figure. S4** N_2_ adsorption-desorption isotherms of (a)CeO_2_, (b)v-CeO_2_, (c)Pt_SA_/v-CeO_2_.


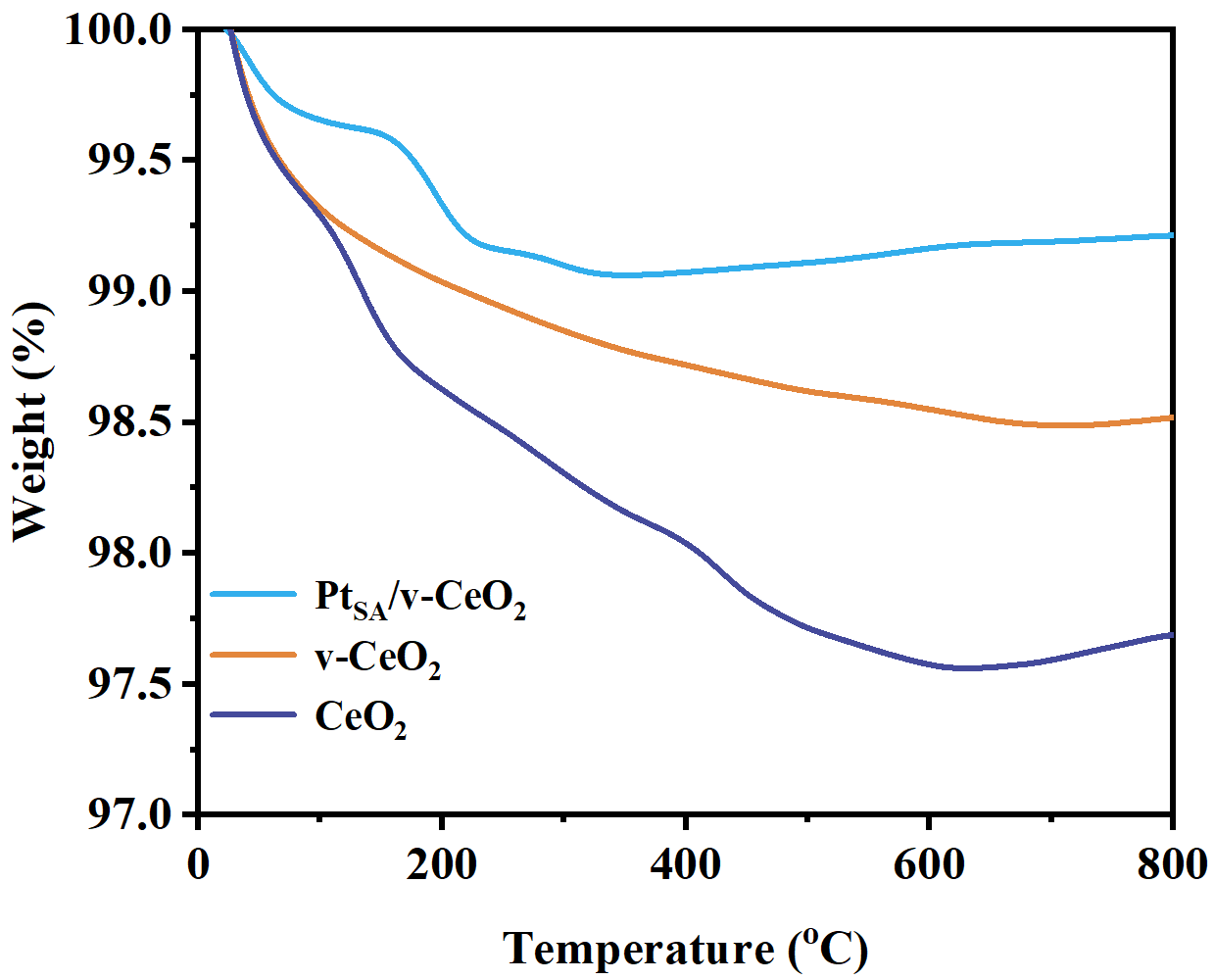


**Figure. S5** TG analysis of CeO_2_, v-CeO_2_ and Pt_SA_/v-CeO_2_.


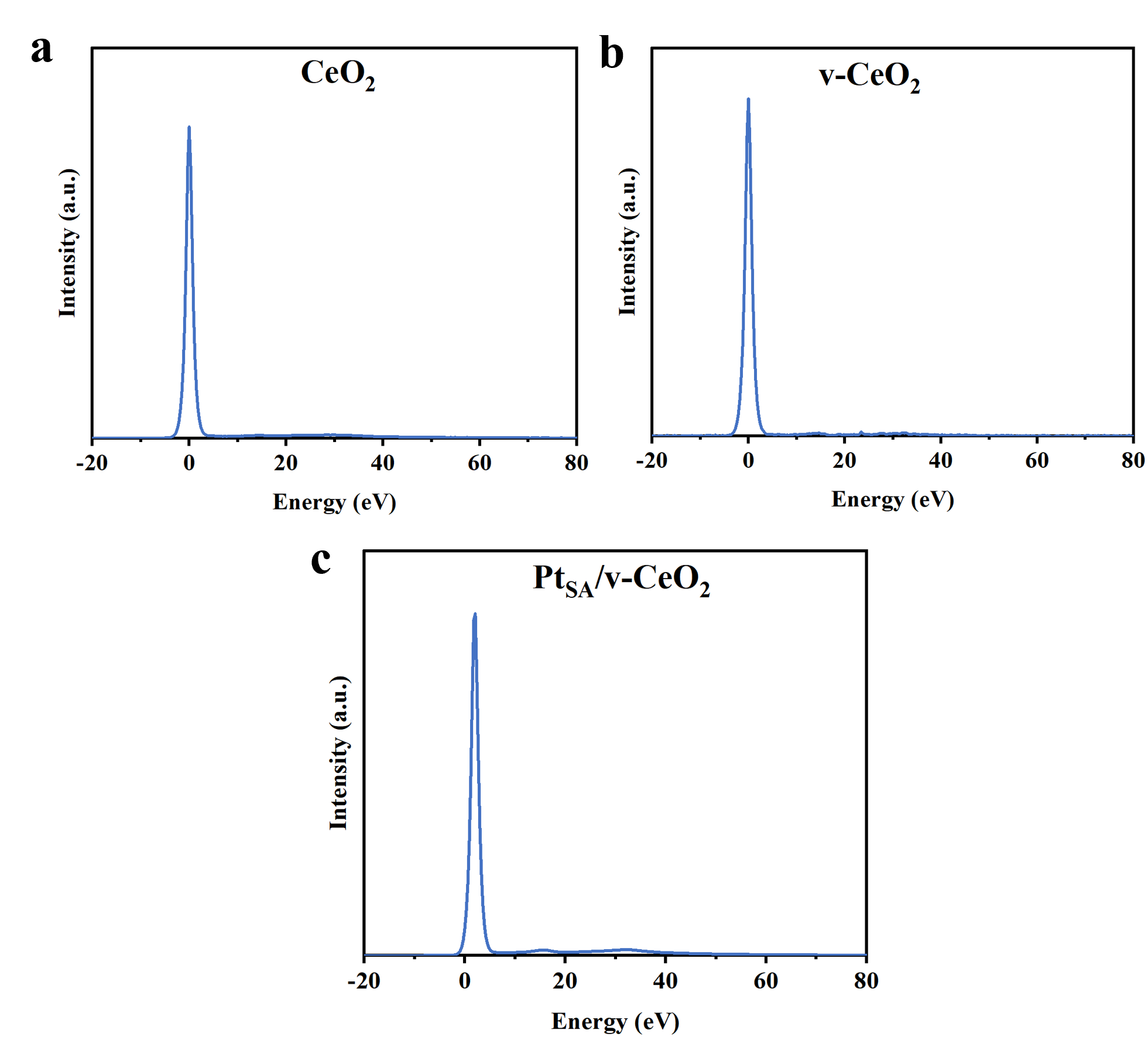


**Fig. S6** Zero-loss peak of (a) CeO_2_, (b) v-CeO_2_, (c) Pt_SA_/v-CeO_2_.

.


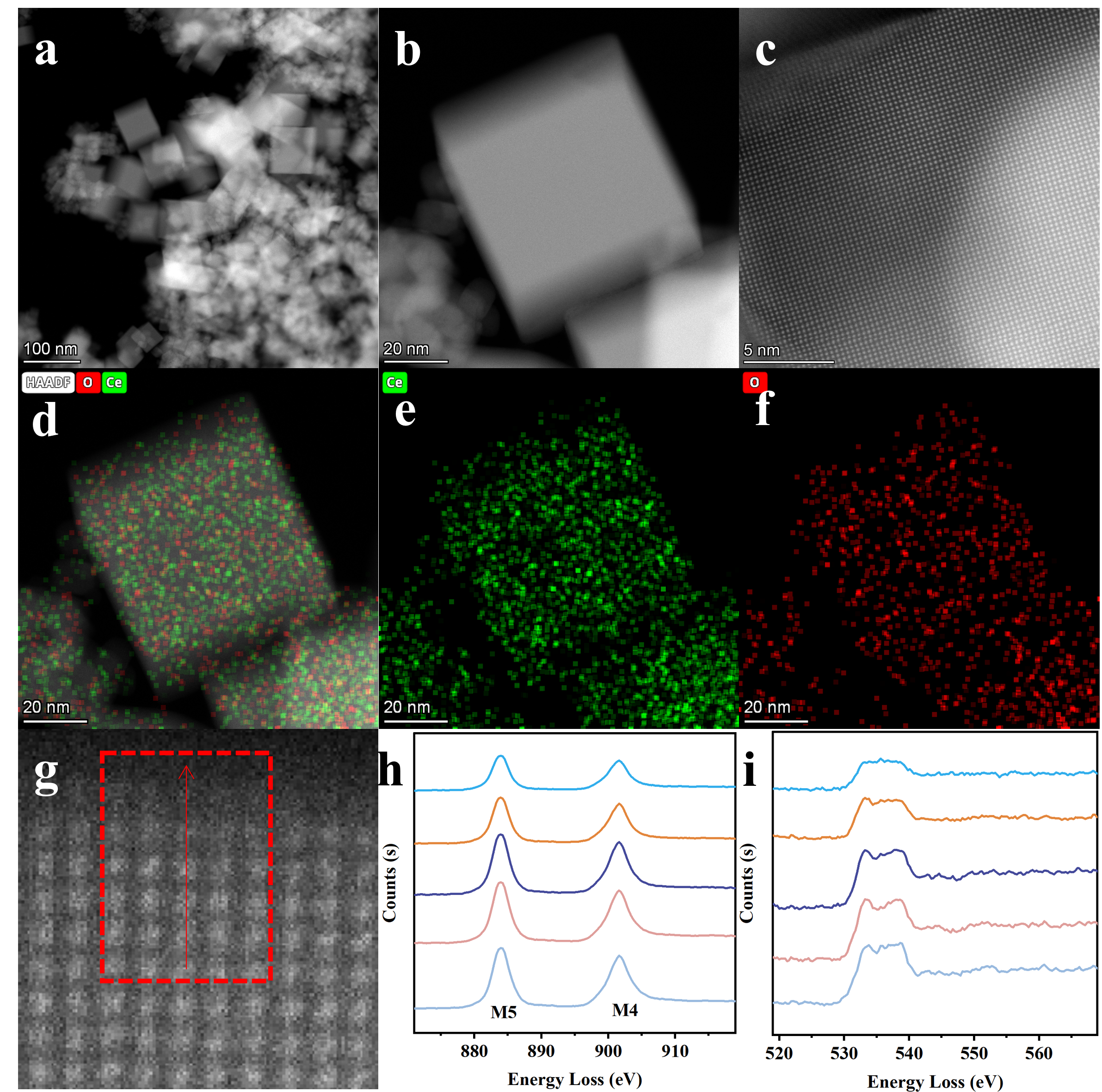


**Fig. S7** (a-c) HAADF image of CeO_2_, (d-f) EDS mapping of CeO_2_, (g-i) EELS analysis of CeO_2_.


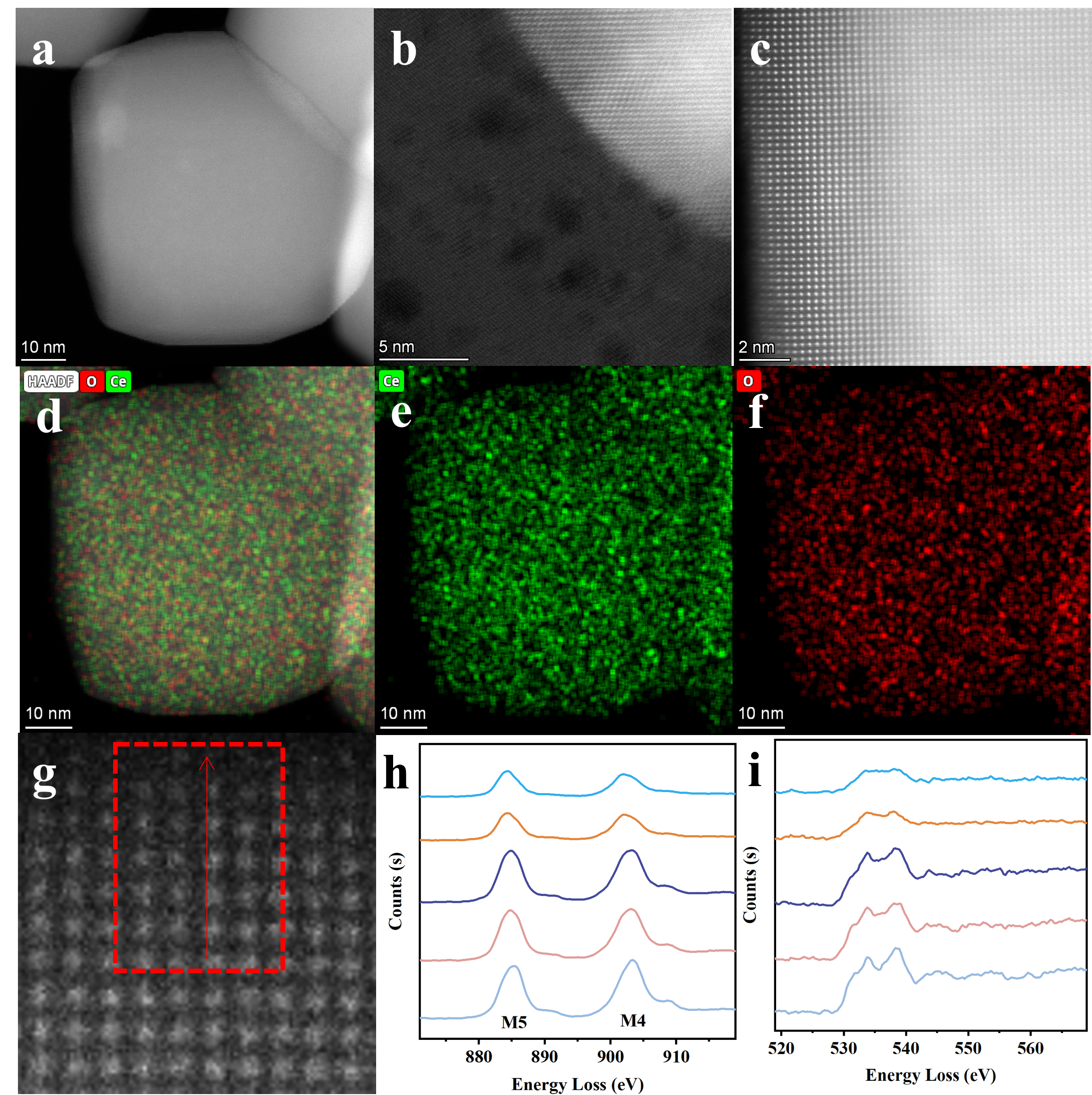


**Fig. S8** (a-c) HAADF image of v-CeO_2_, (d-f) EDS mapping of v-CeO_2_, (g-i) EELS analysis of v-CeO_2_.


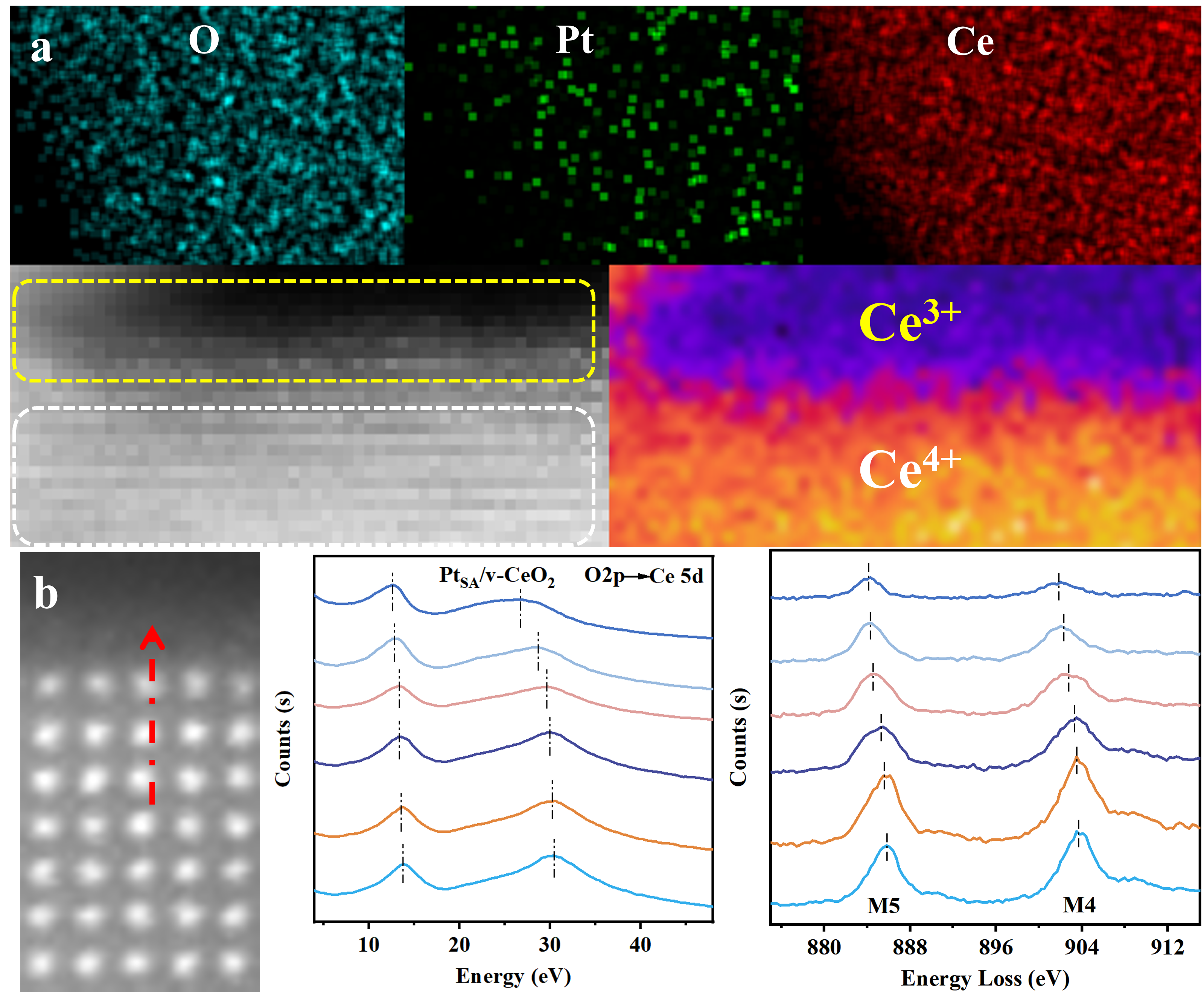


**Fig. S9** (a) EDS analysis of Pt_SA_/v-CeO_2_. (b) atomic resolved monochromatic EELS spectroscopy of Pt_SA_/v-CeO_2_ surface atoms.


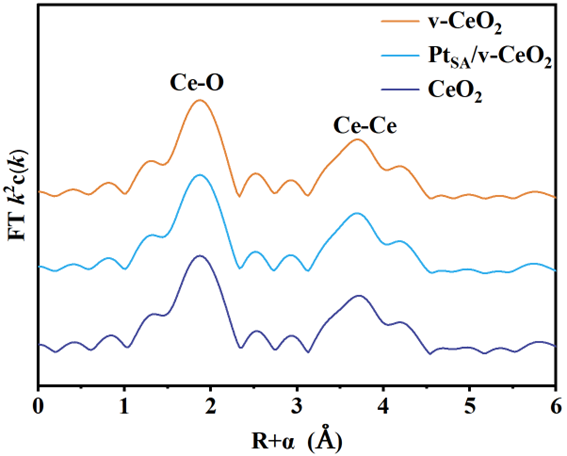


**Fig. S10** Ce L_3_-edge Fourier-transformed EXAFS spectra of CeO_2_, v-CeO_2_ and Pt_SA_/v-CeO_2_. R denotes the bonding distance of chemical bonds.


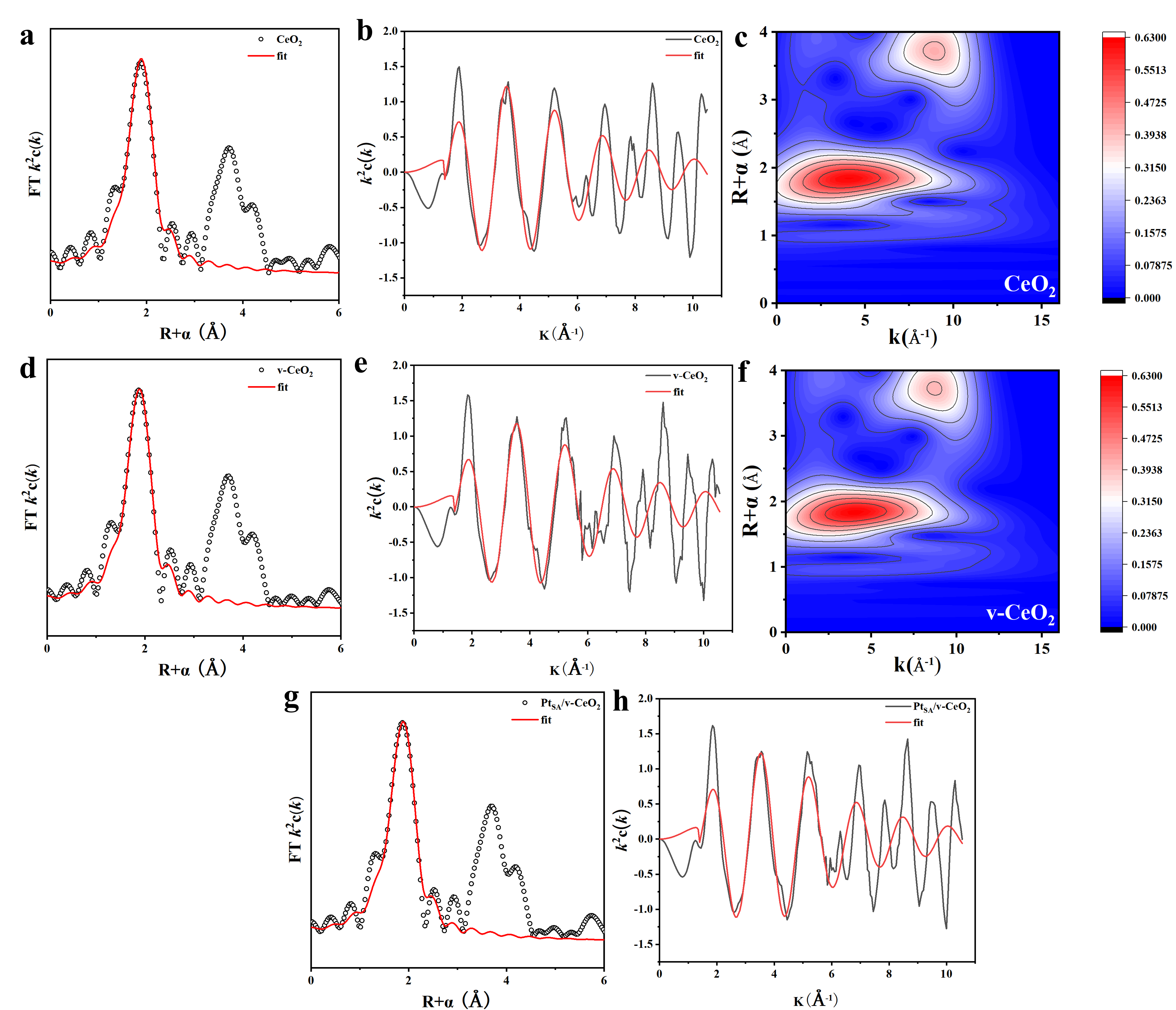


**Fig. S11** FT-EXAFS curves at Ce L_3_-edge, where the curve is experimental data and the circle is the best fit for (a) CeO_2_, (d) v-CeO_2_ and (g) Pt_SA_/v-CeO_2_, Experimental and fitting results of Fourier transformed extended X-ray absorption fine structure (EXAFS) spectra of (b) CeO_2_, (e) v-CeO_2_ and (h) Pt_SA_/v-CeO_2_, WT-EXAFS signals at Ce L_3_-edge for (c) CeO_2_ and (f) v-CeO_2_.


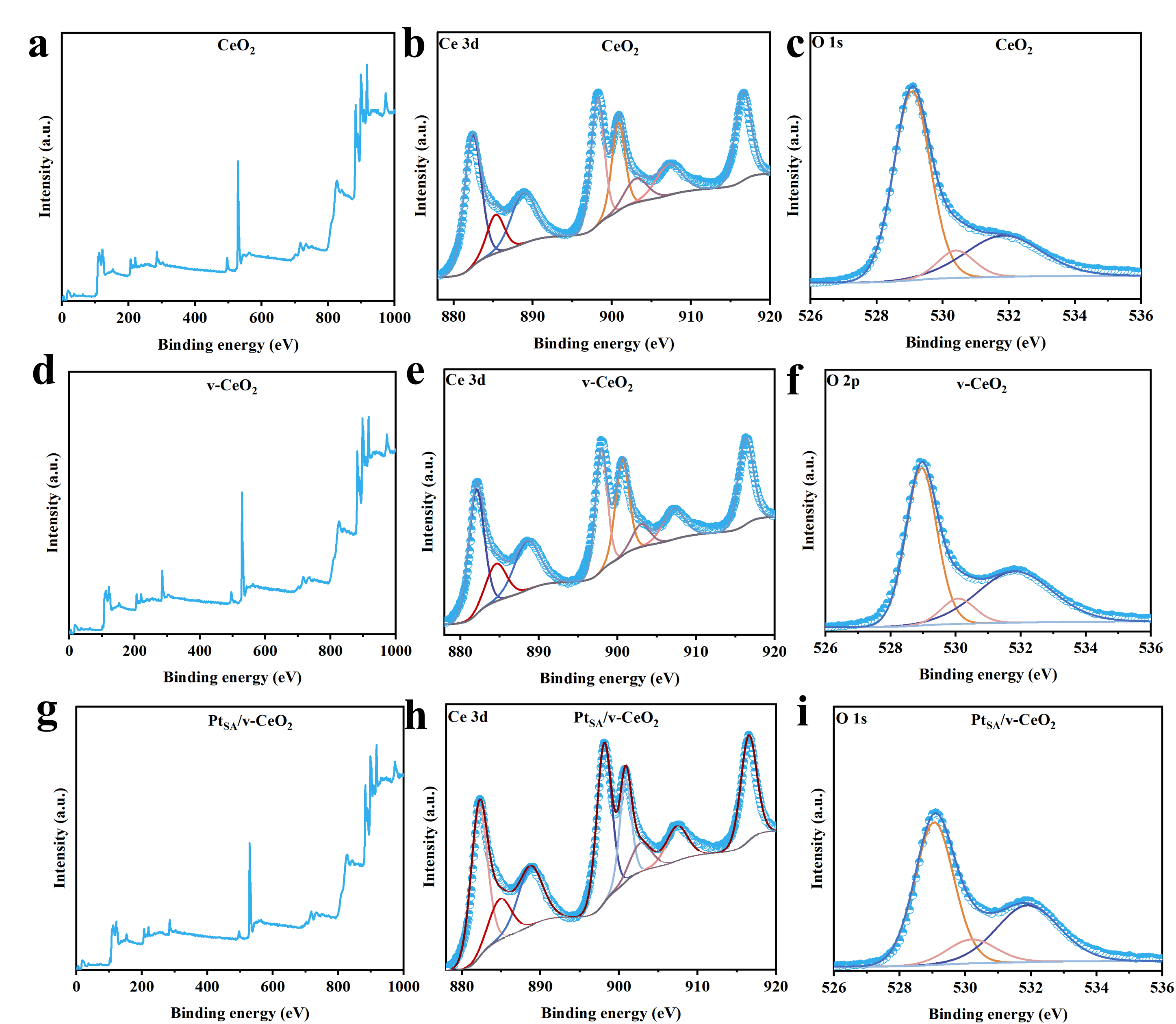


**Fig. S12** Ce 3d and O 2p high-resolution XPS spectra of (a-c) CeO_2_, (d-f) v-CeO_2_, (g-i) Pt_SA_/v-CeO_2_.


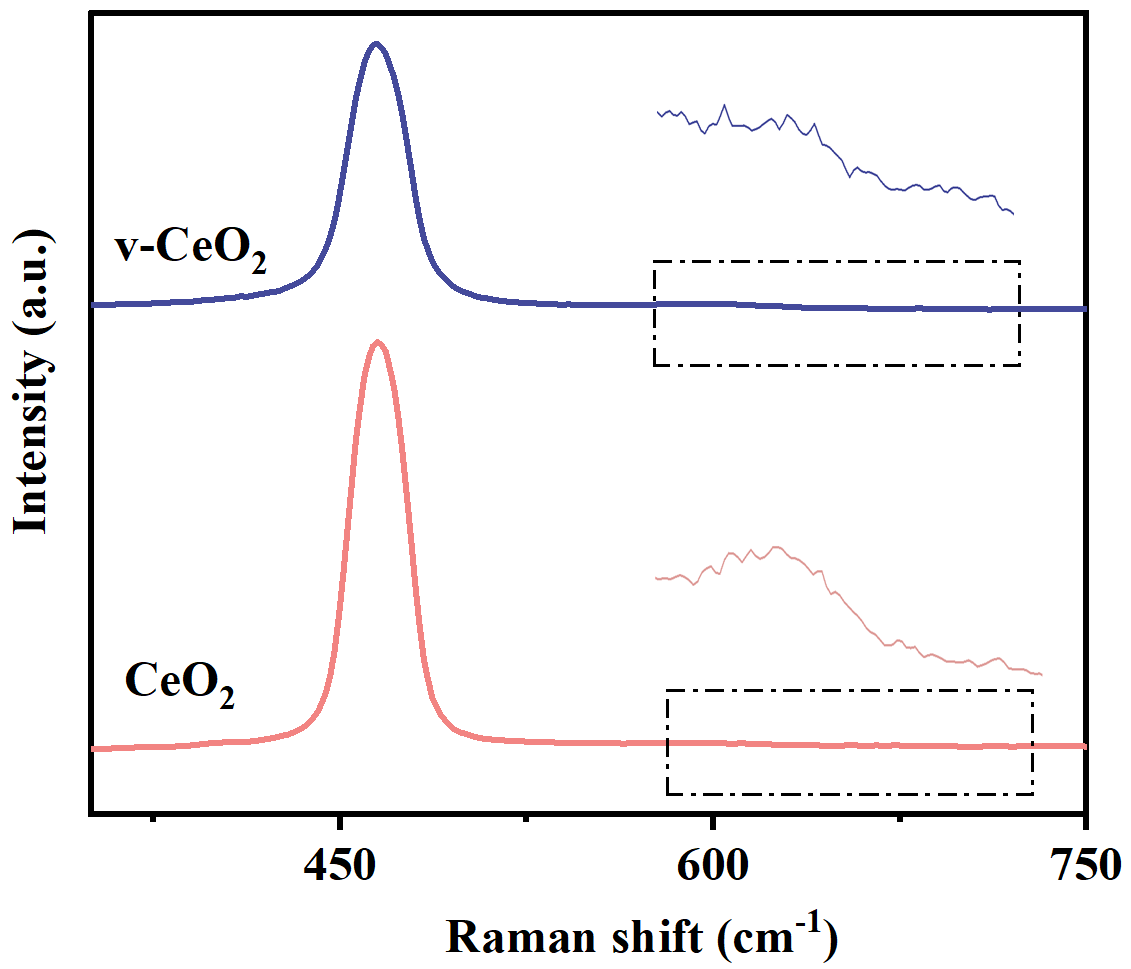


**Fig. S13** The Raman spectra of CeO_2_ and v-CeO_2_.


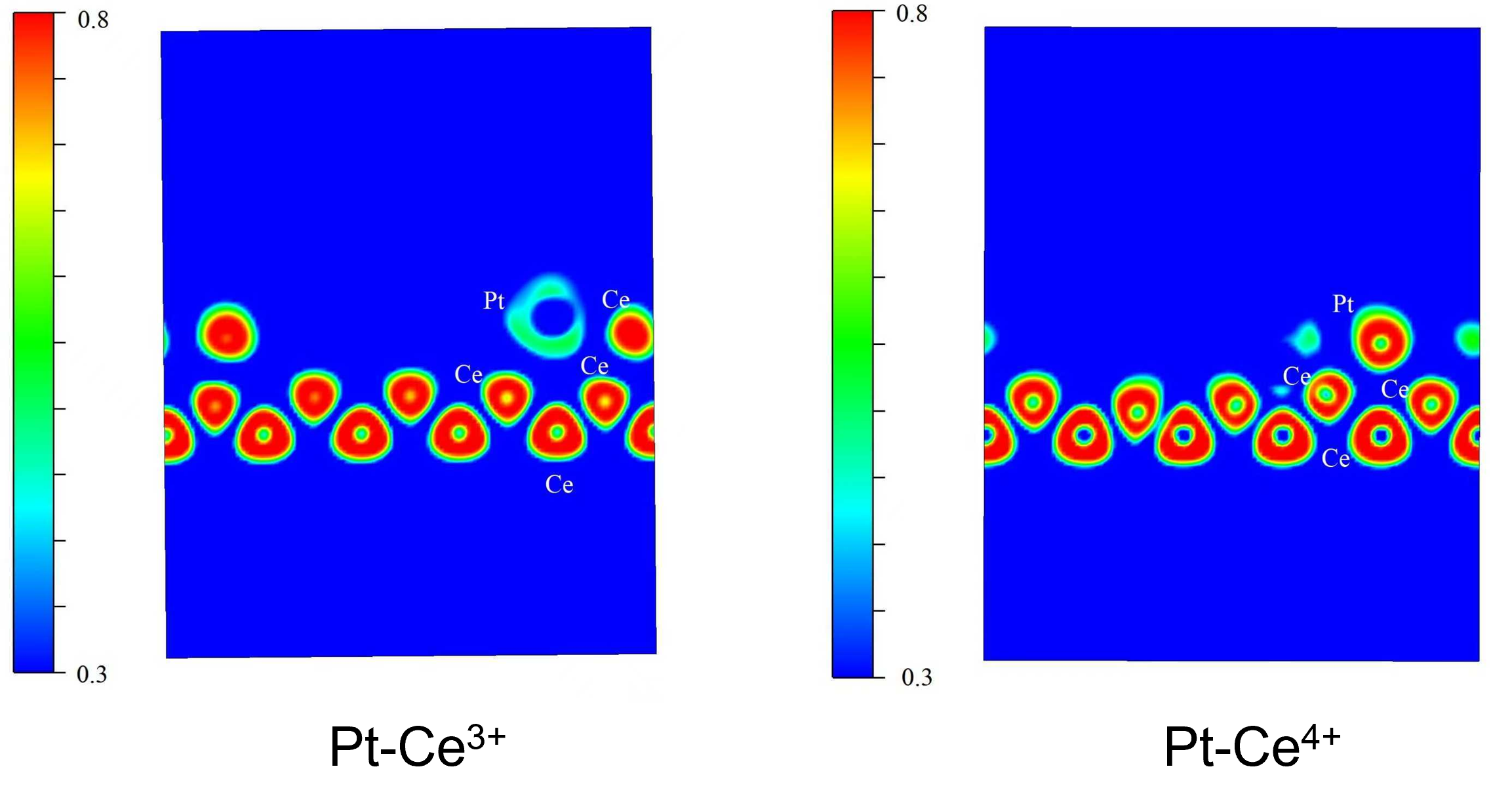


**Fig.14** Potential gradient analysis of Pt_SA_-Ce^3+^ and Pt_SA_-Ce^4+^


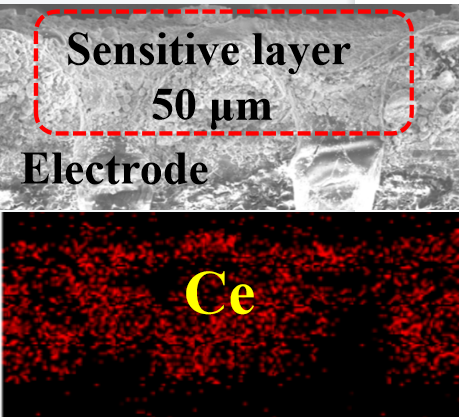

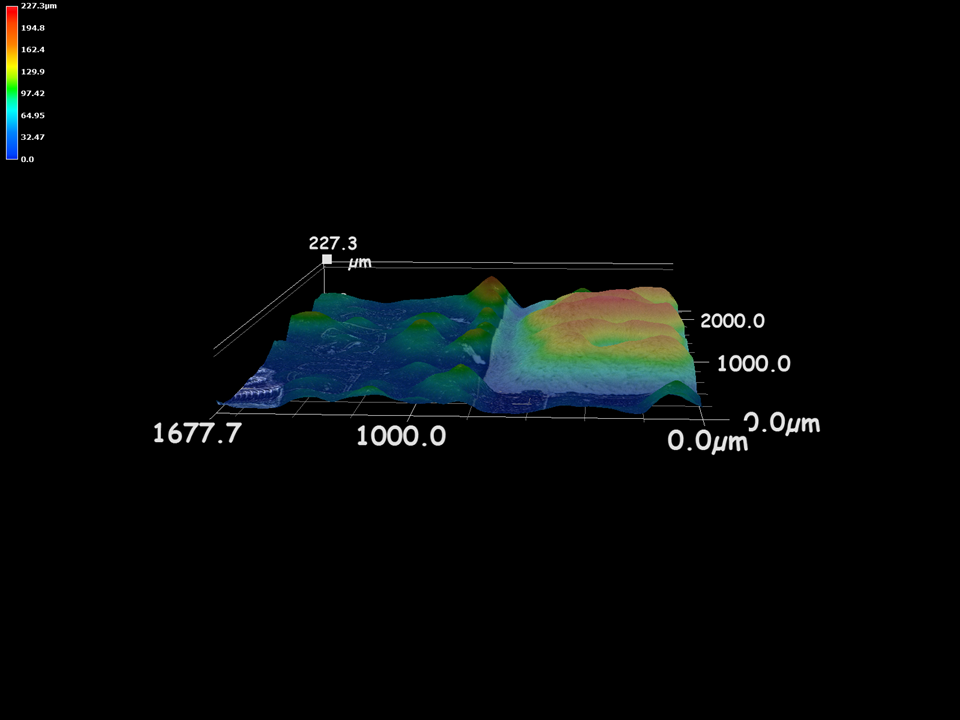


**Fig. S15** The thickness of sensing layer.


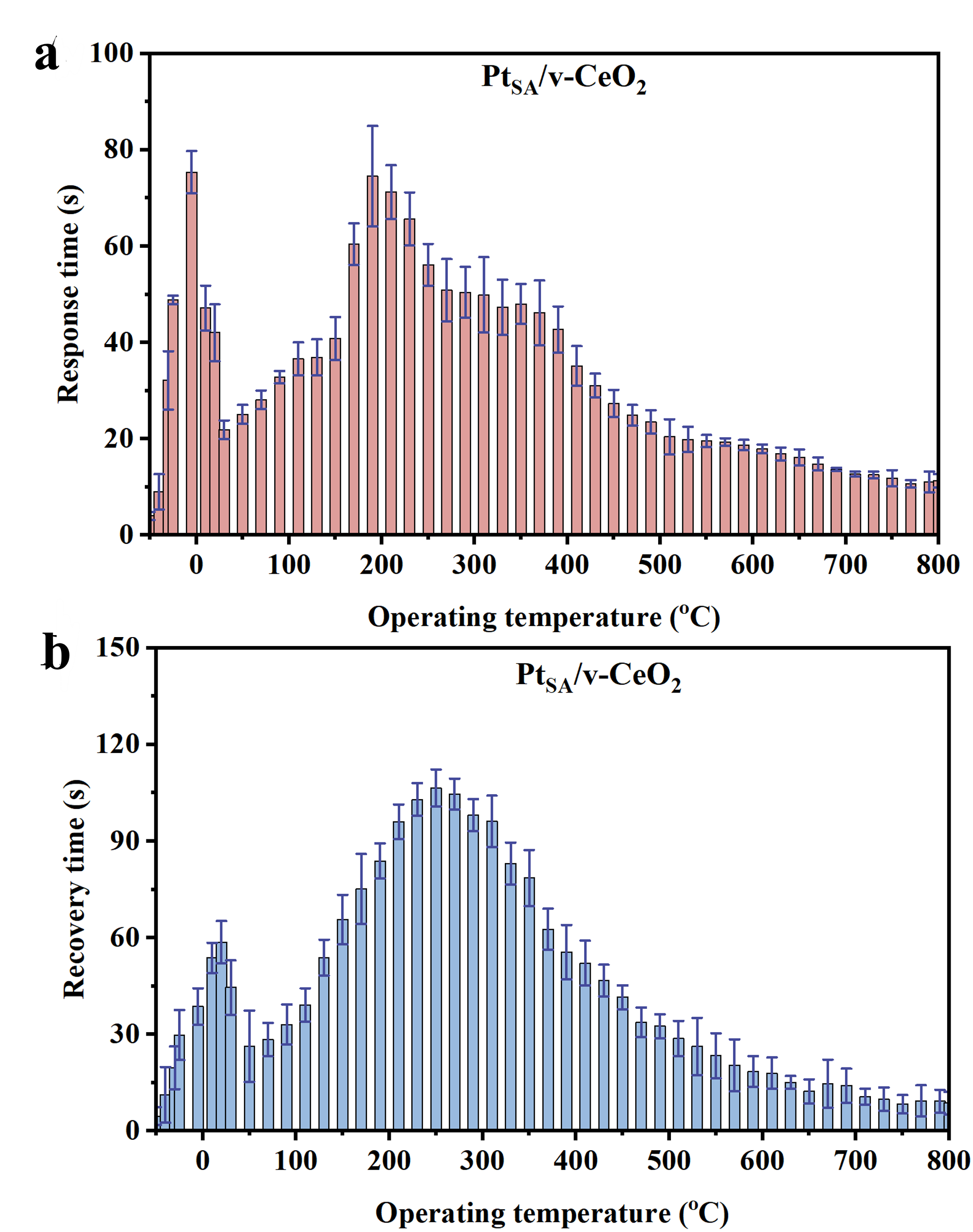


**Fig. S16** Response-recovery time of Pt_SA_/v-CeO_2_.


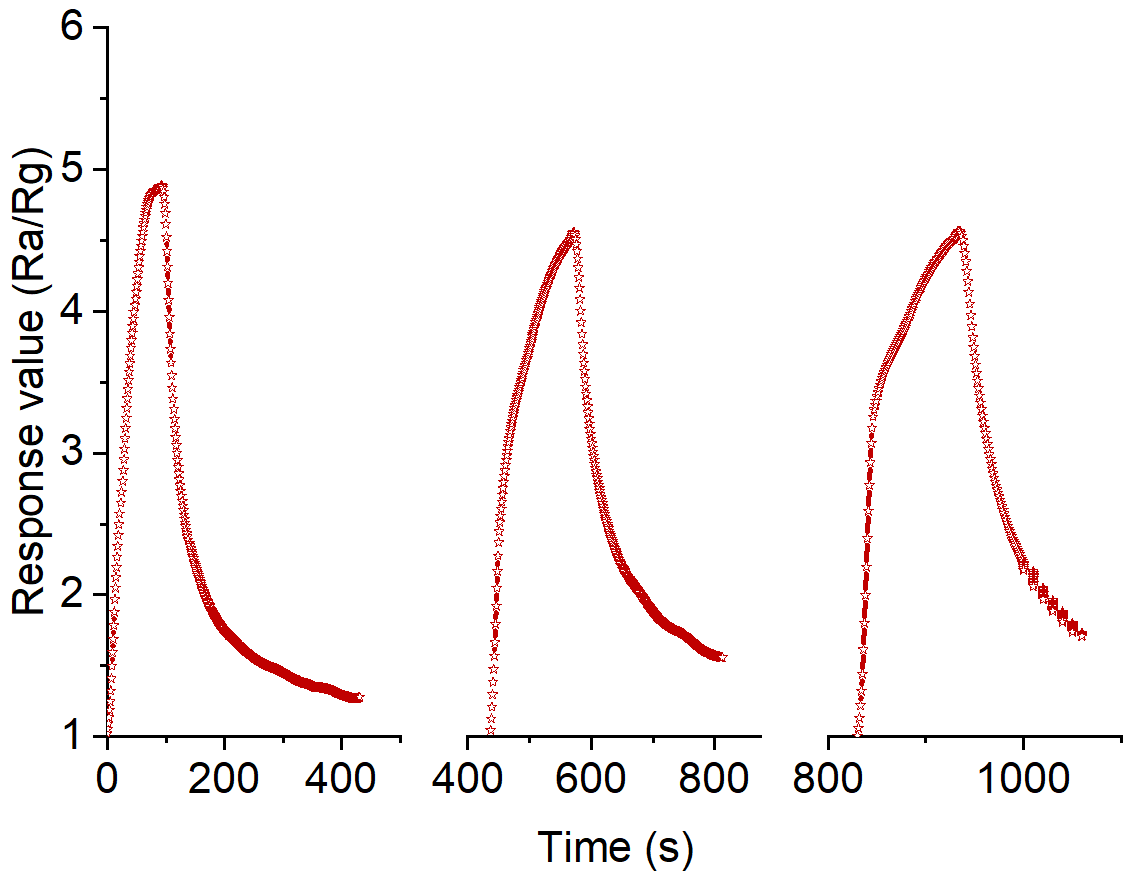


**Fig.S17**  The cycle stability test of Pt_SA_/v-CeO_2_ at 200^o^C


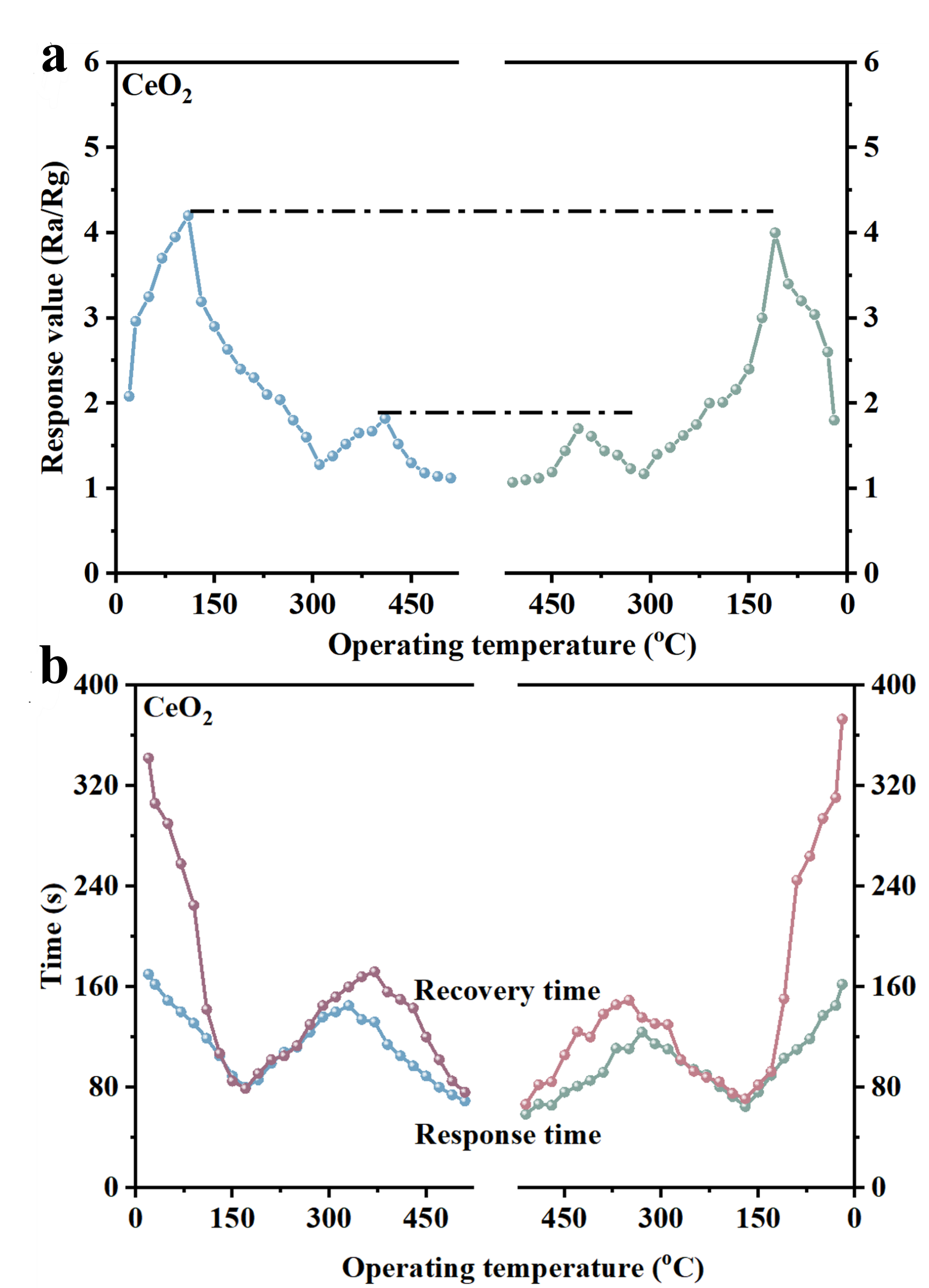


**Fig. S18** The response value of (a) CeO_2_, (b) Response-recovery time of CeO_2_.


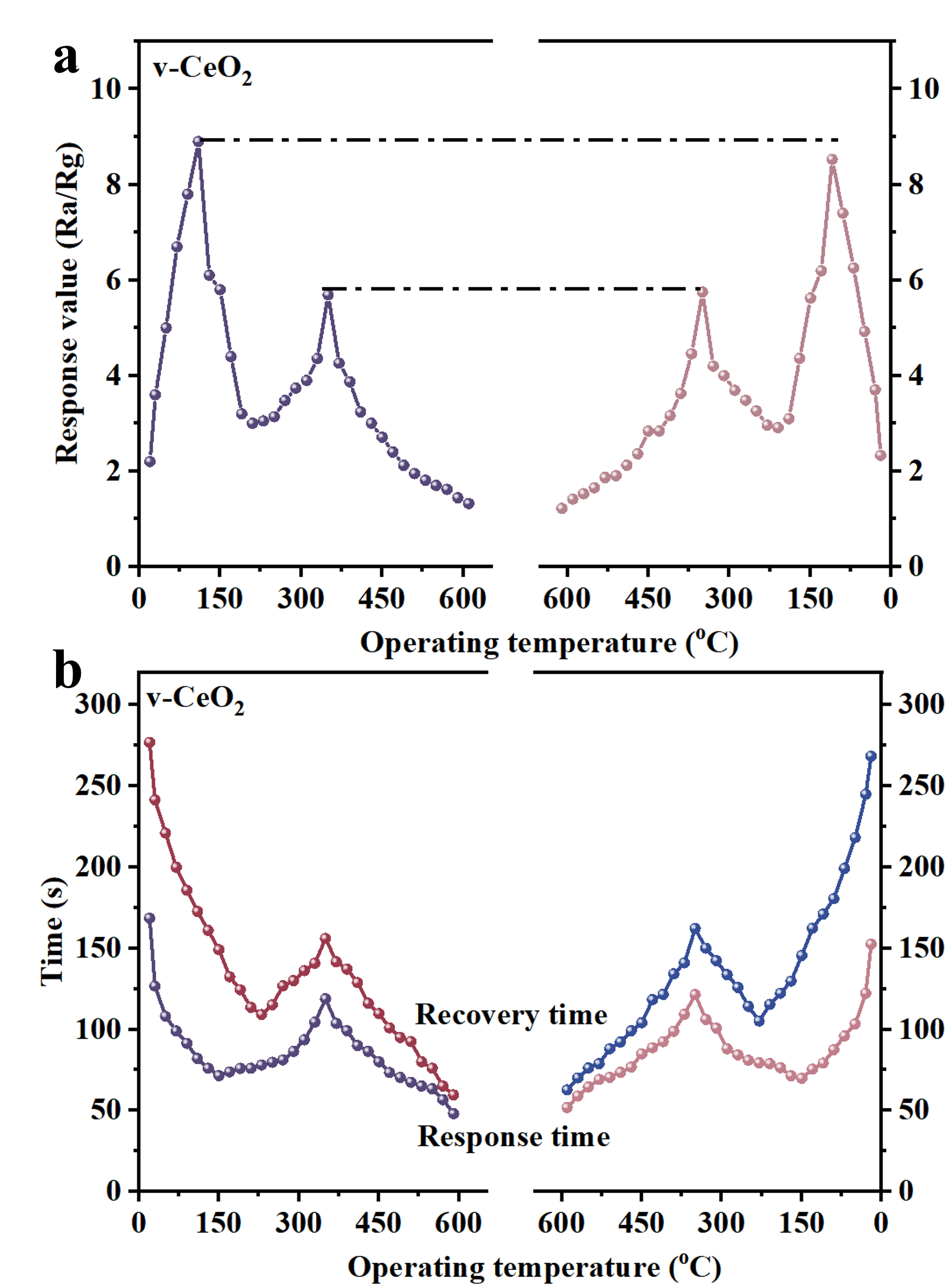


**Fig. S19** The response value of (a) v-CeO_2_, (b) Response-recovery time of v-CeO_2_.


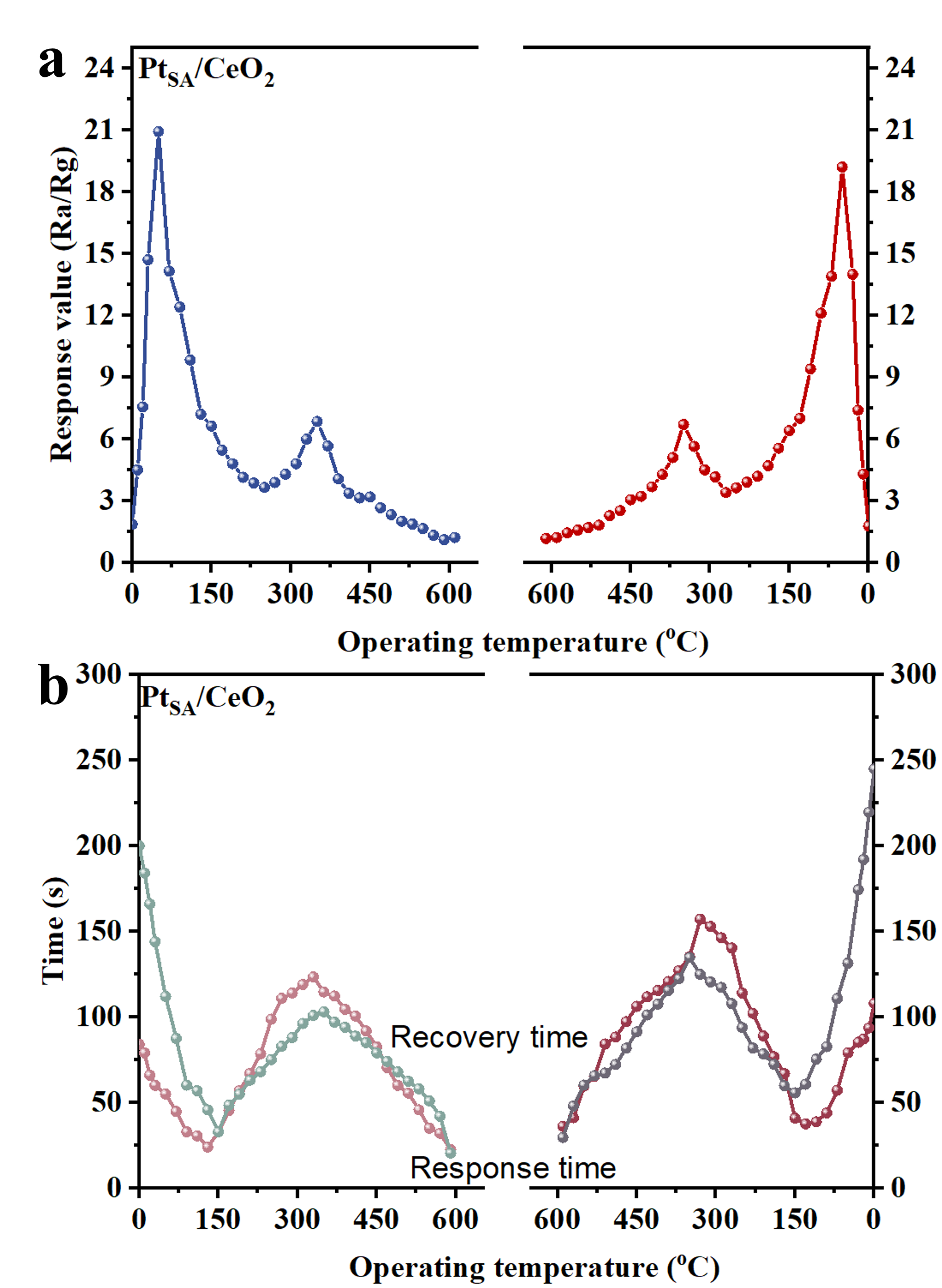


**Fig. S20** The response value of (a) Pt_SA_/CeO_2_, (b) Response-recovery time of Pt_SA_/CeO_2_.


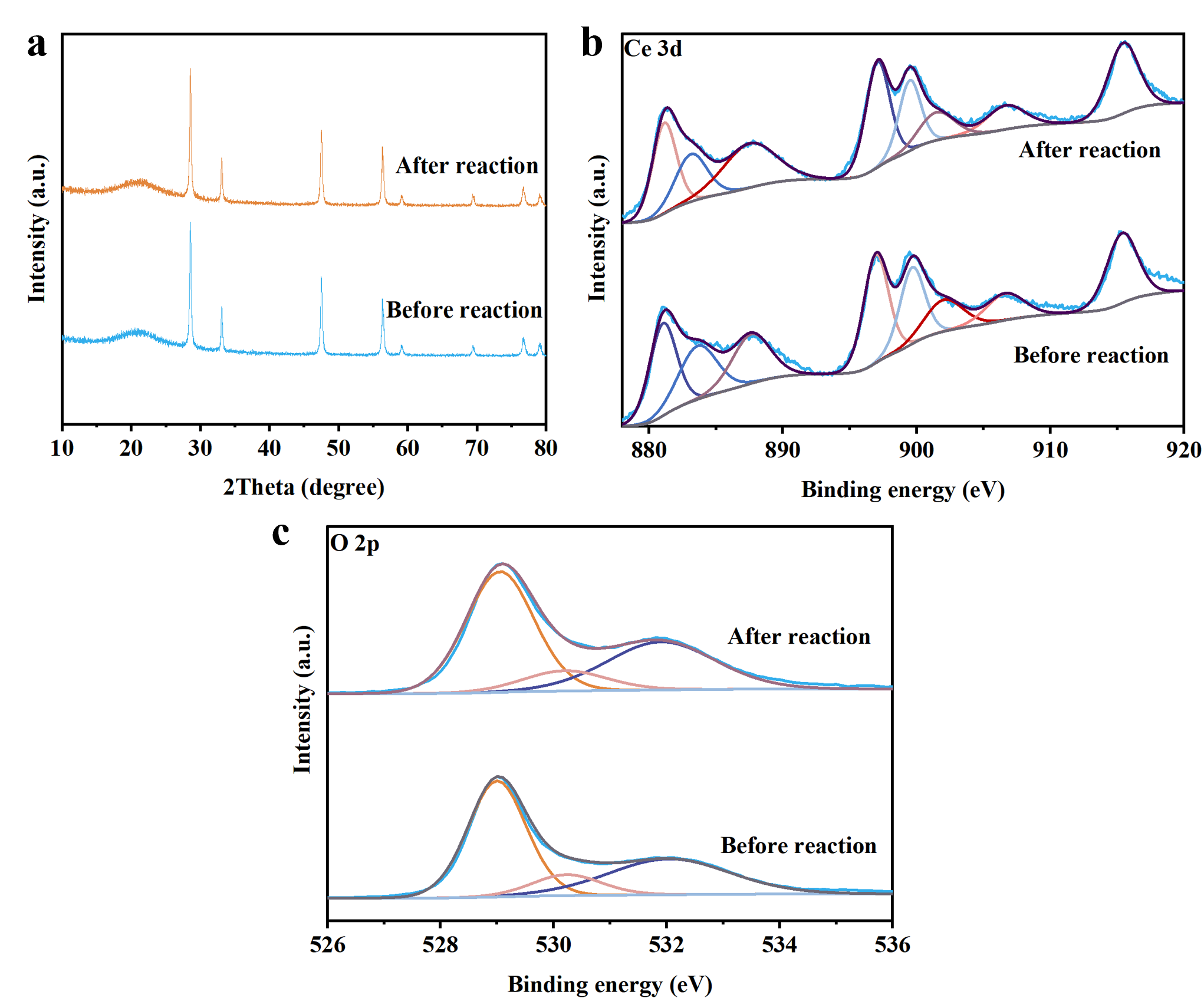


**Fig. S21** (a) XRD pattern of Pt_SA_/v-CeO_2_ after reaction, (b-c) Ce 3d and O2p high-resolution XPS spectra of Pt_SA_/v-CeO_2_ after reaction CeO_2_.


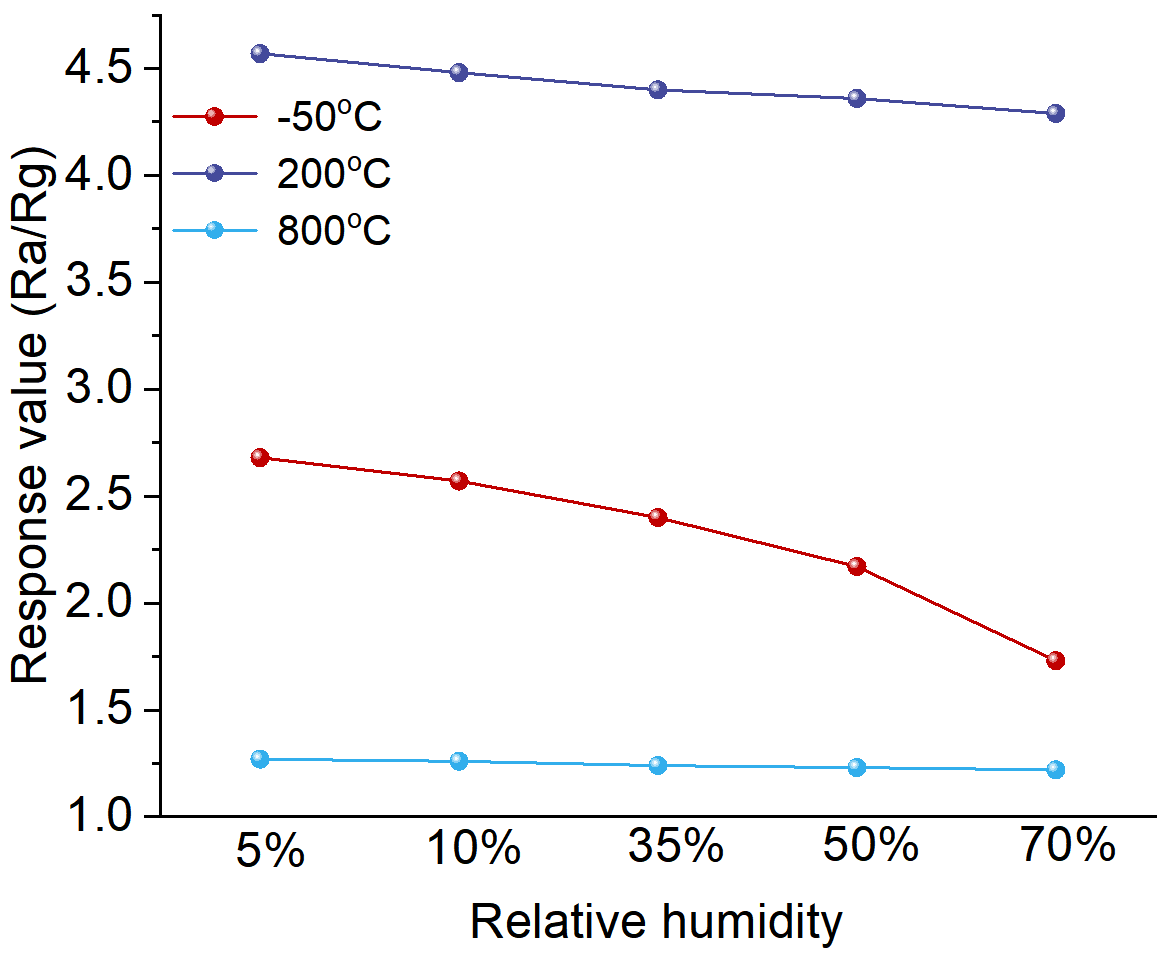


**Fig.S22**  The gas-sensing performance of Pt_SA_/v-CeO_2_ under different relative humidity


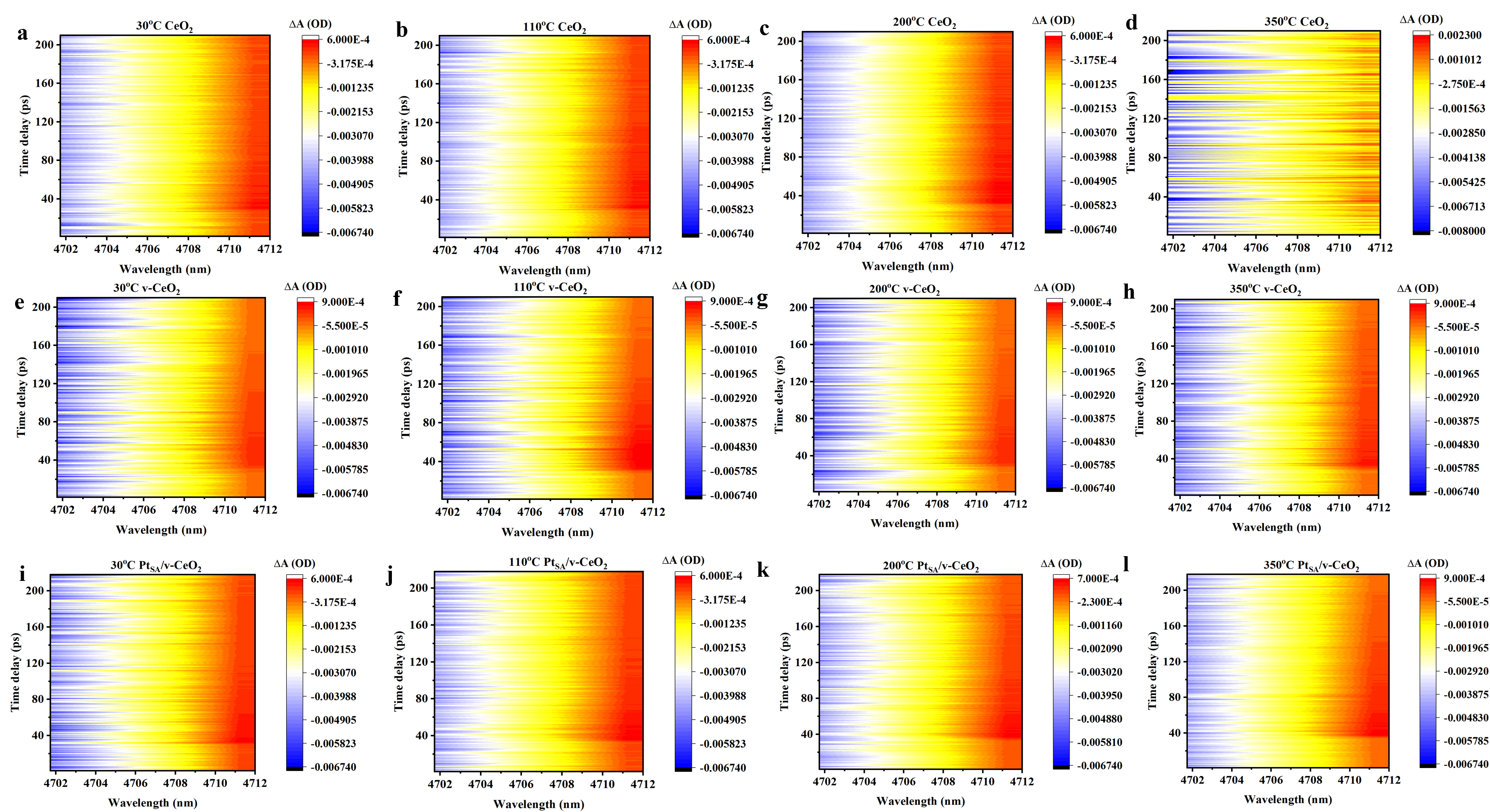


**Fig. S23** 2D pseudo-color Temperature-dependent of MIR-TAS maps of (a-d) CeO_2_, (e-h) v-CeO_2_, (i-l) Pt_SA_/v-CeO_2_.

**
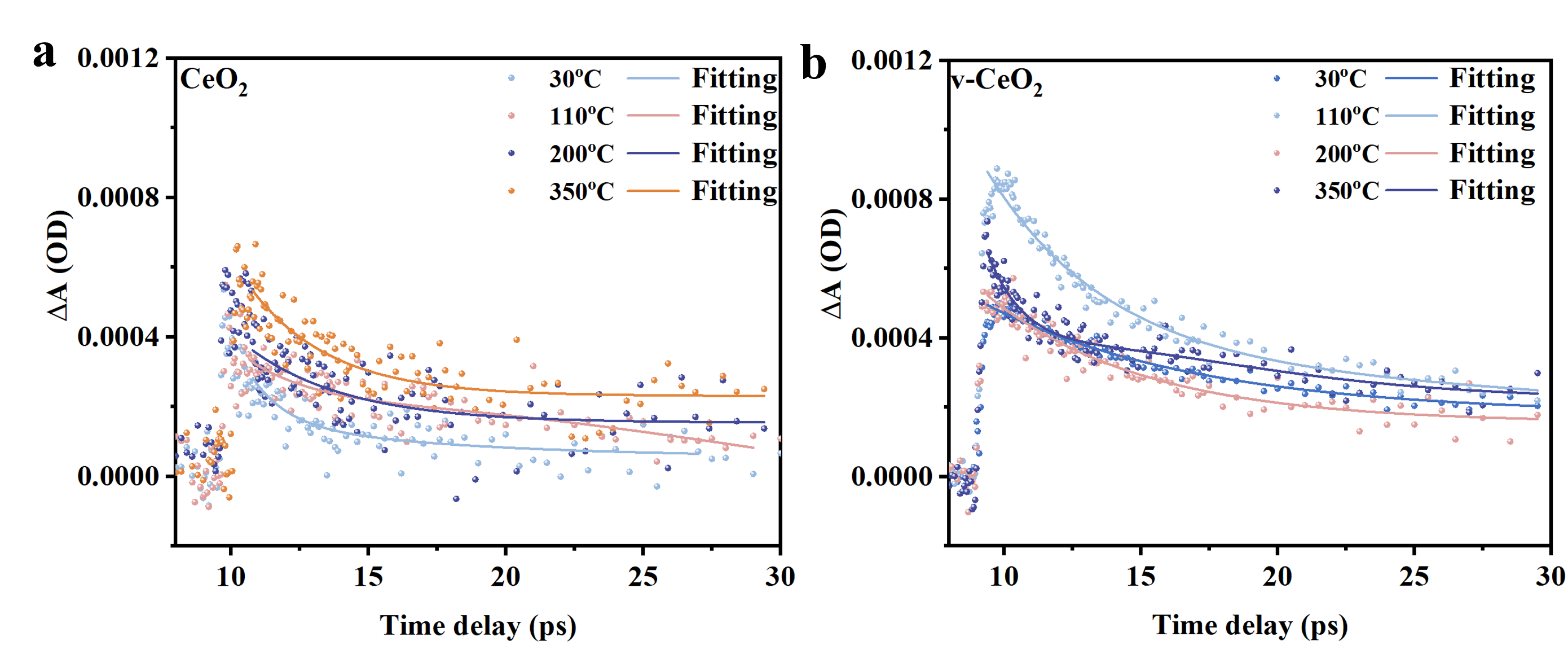
**

**Fig. S24** Temperature-dependent MIR-TAS of (a) CeO_2_ and (b) v-CeO_2_.


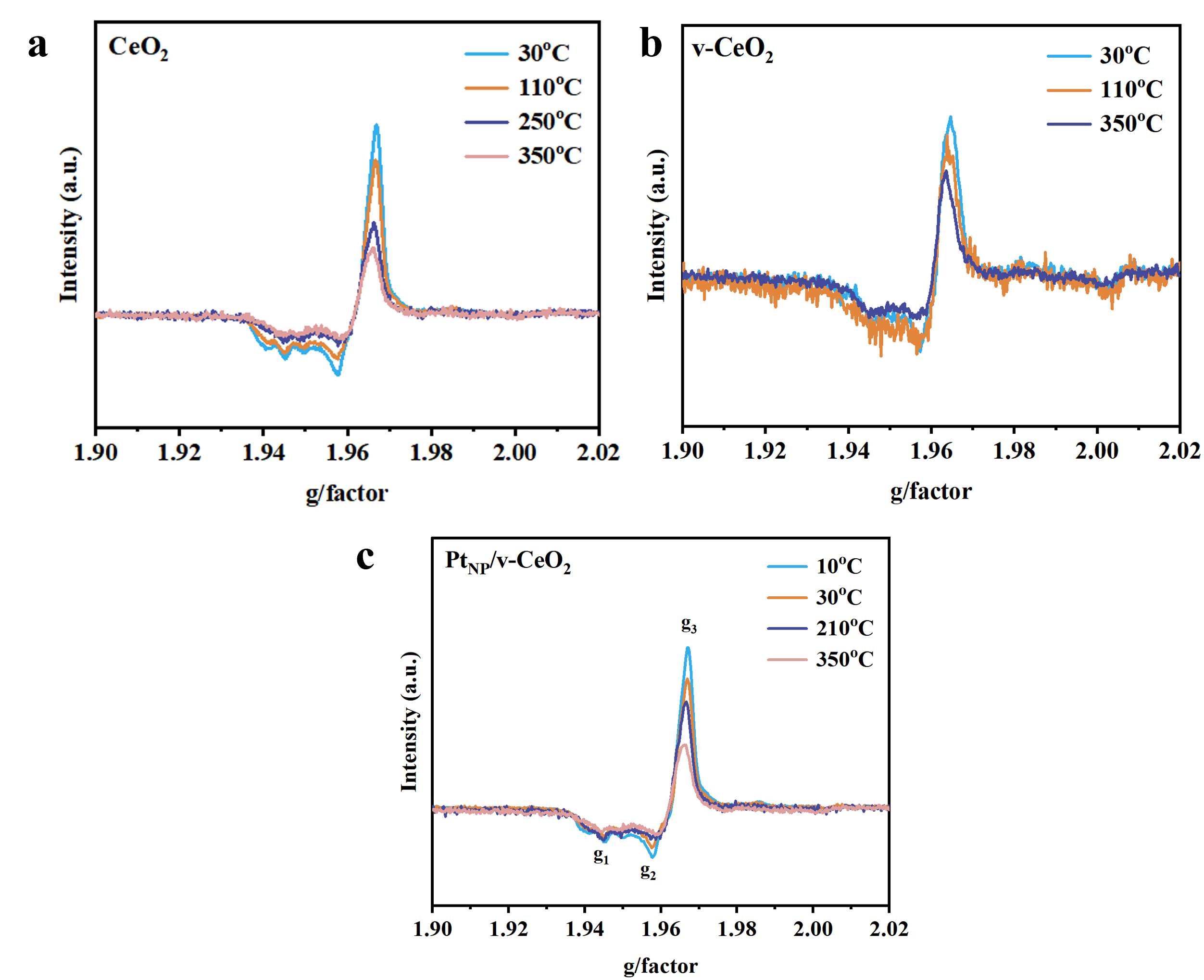


**Fig. S25** (a) In-situ EPR analysis of CeO_2_ under different temperature, (b) In-situ EPR analysis of v-CeO_2_ under different temperature.


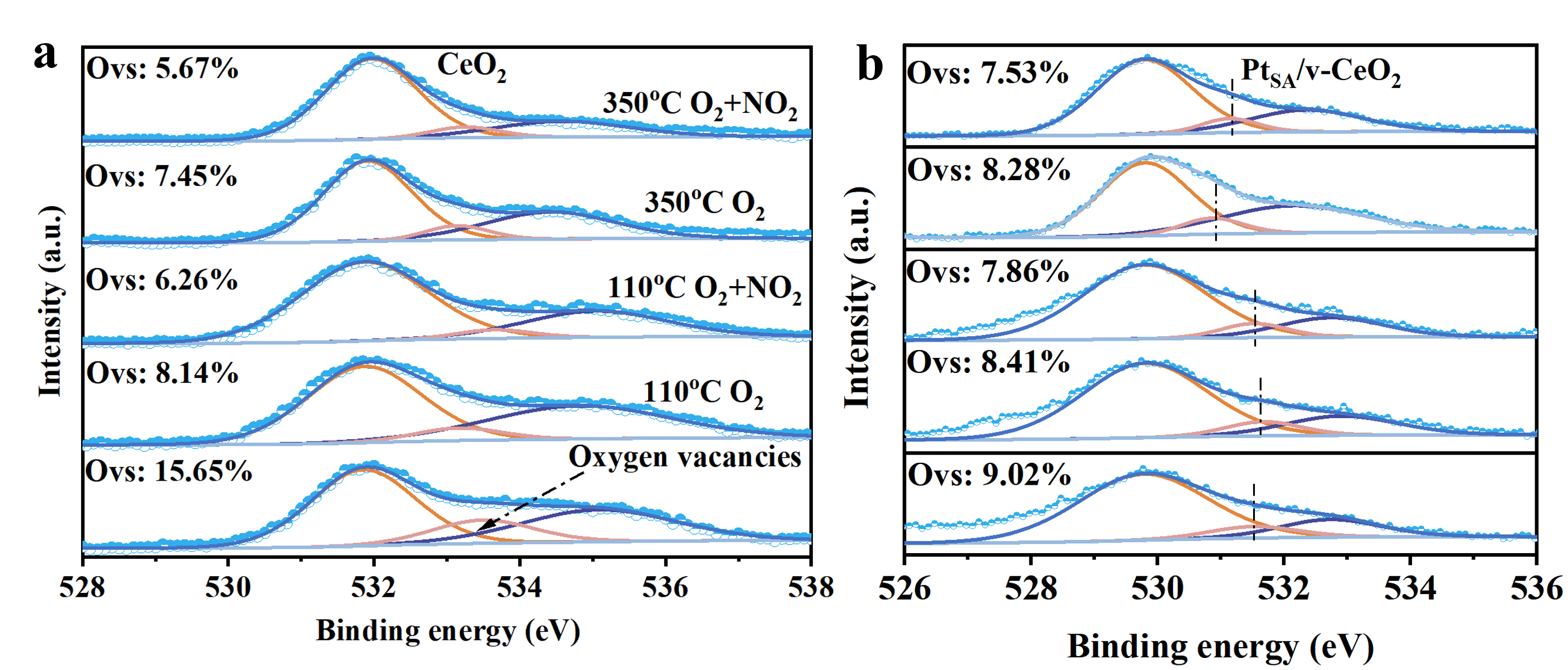


**Fig. S26** In-situ NAP-XPS analysis of CeO_2_ and Pt_SA_/v-CeO_2_ at different temperature under O_2_ and NO_2_


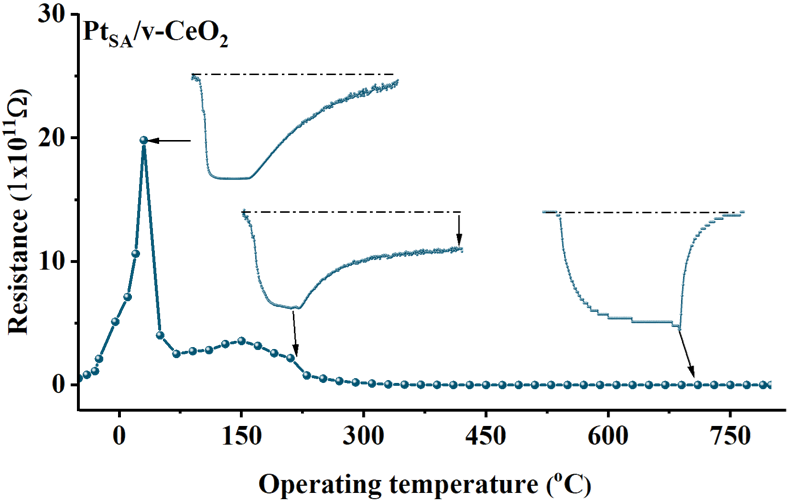


**Fig. S27** Resistance of Pt_SA_/v-CeO_2_ at different temperature


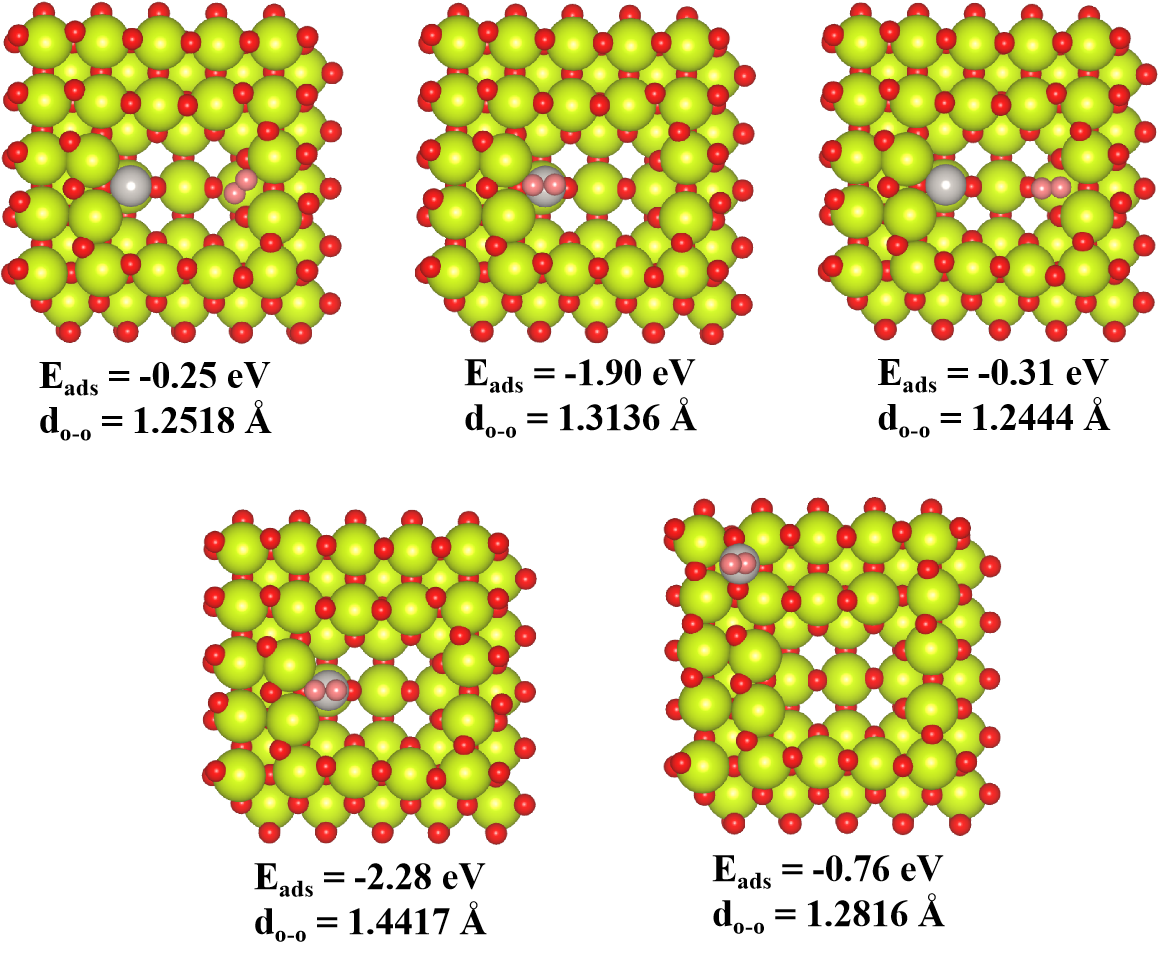


**Fig. S28** The adsorption model of O_2_ in Pt_SA_/v-CeO_2_.


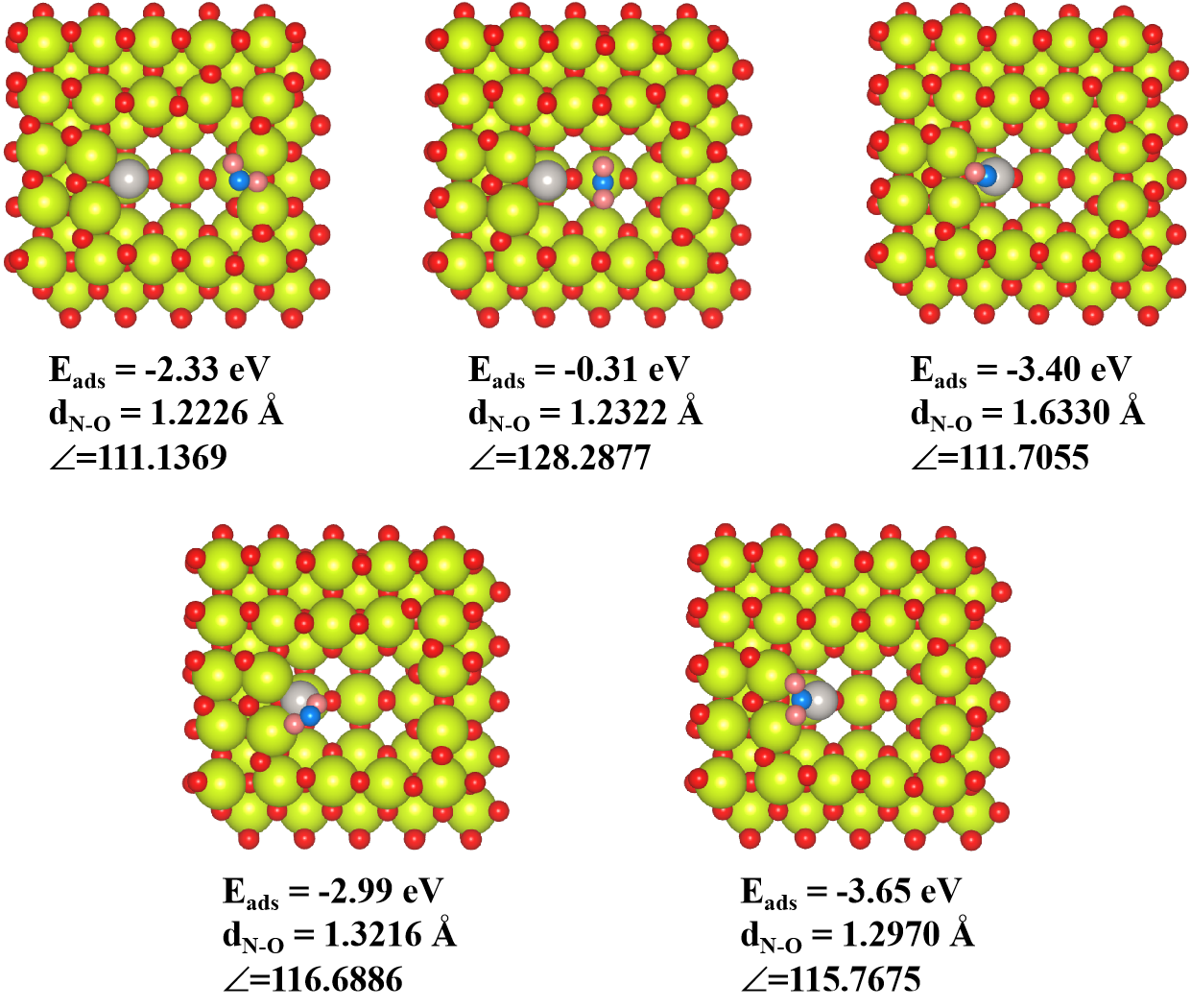


**Fig. S29** The adsorption model of NO_2_ in Pt_SA_/v-CeO_2_.


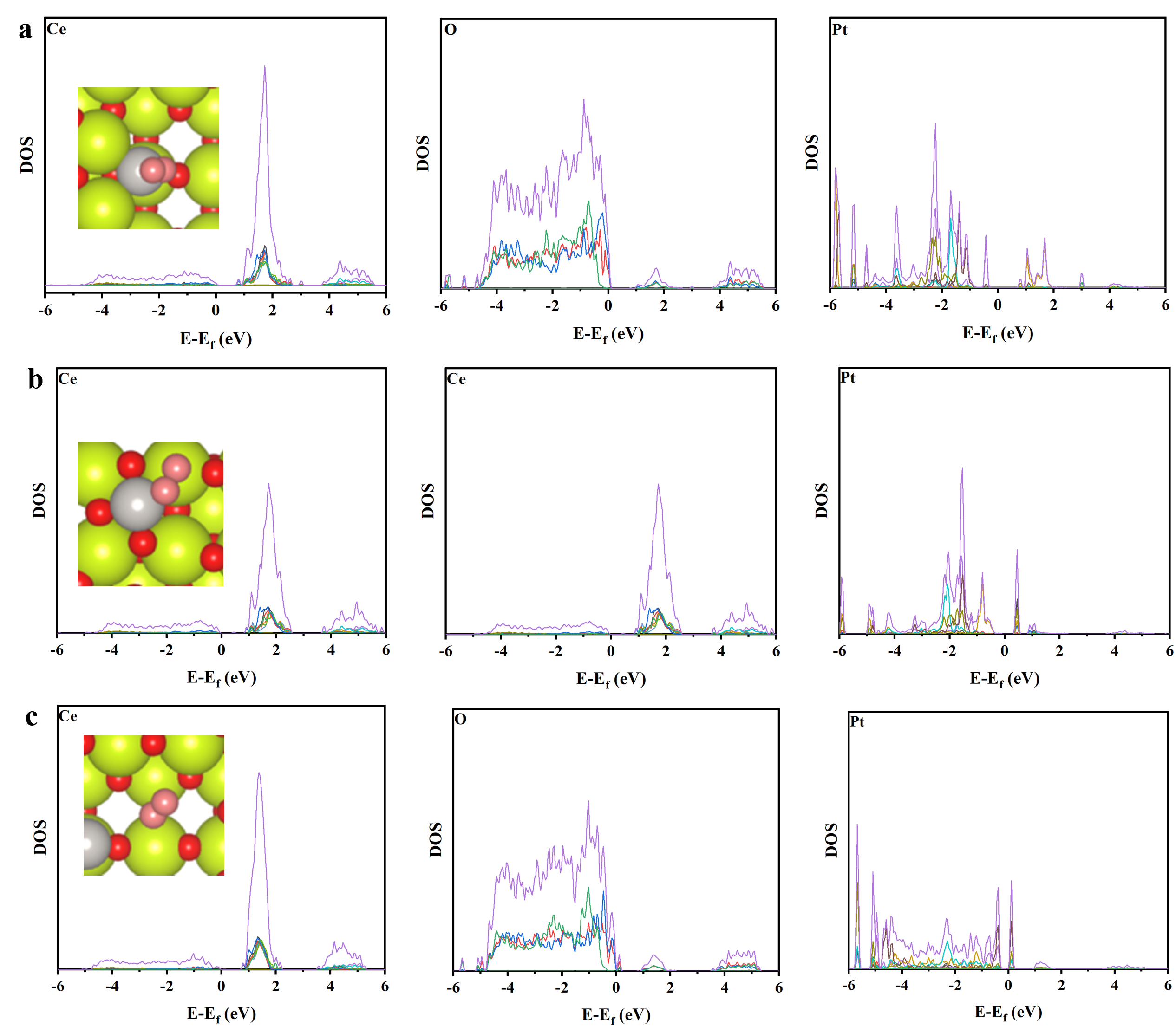


**Fig. S30** DOS of O_2_ in Pt_SA_/v-CeO_2_.


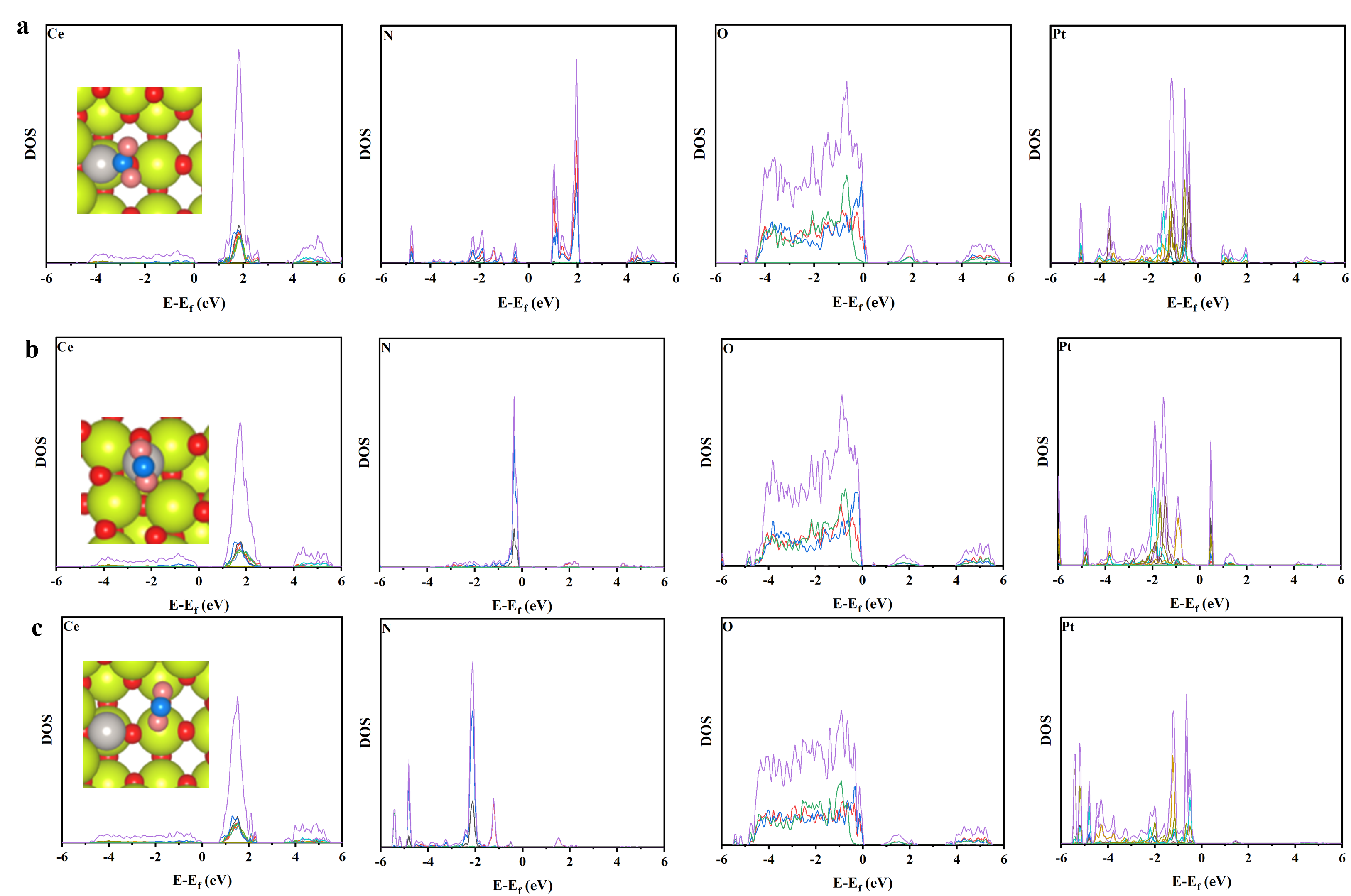


**Fig. S31** DOS of NO_2_ in Pt_SA_/v-CeO_2_.

**
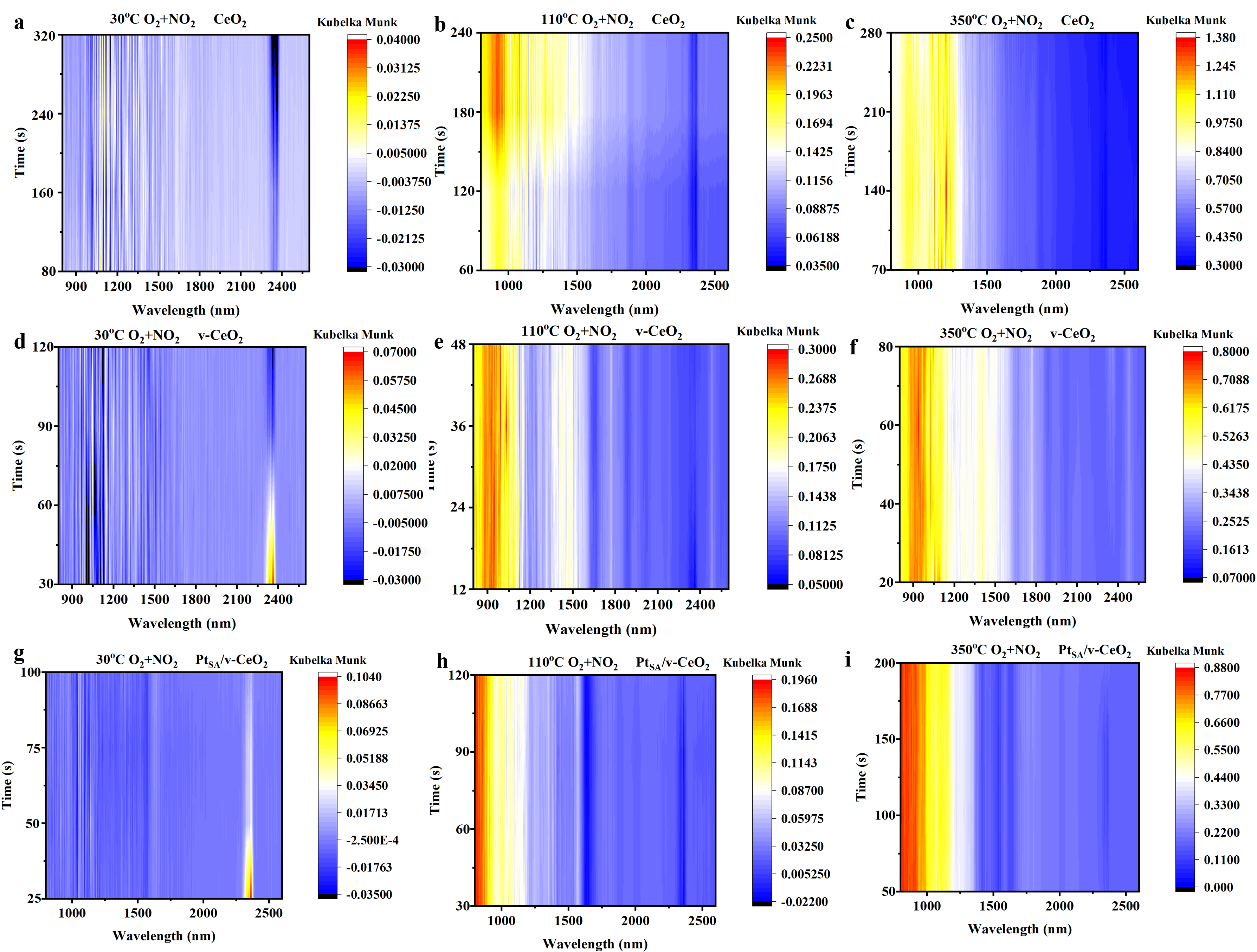
**

**Fig. S32** 2D pseudo-colour in-situ FTIR spectra of (a-c) CeO_2_, (d-f) v-CeO_2_, (g-i) Pt_SA_/v-CeO_2_.


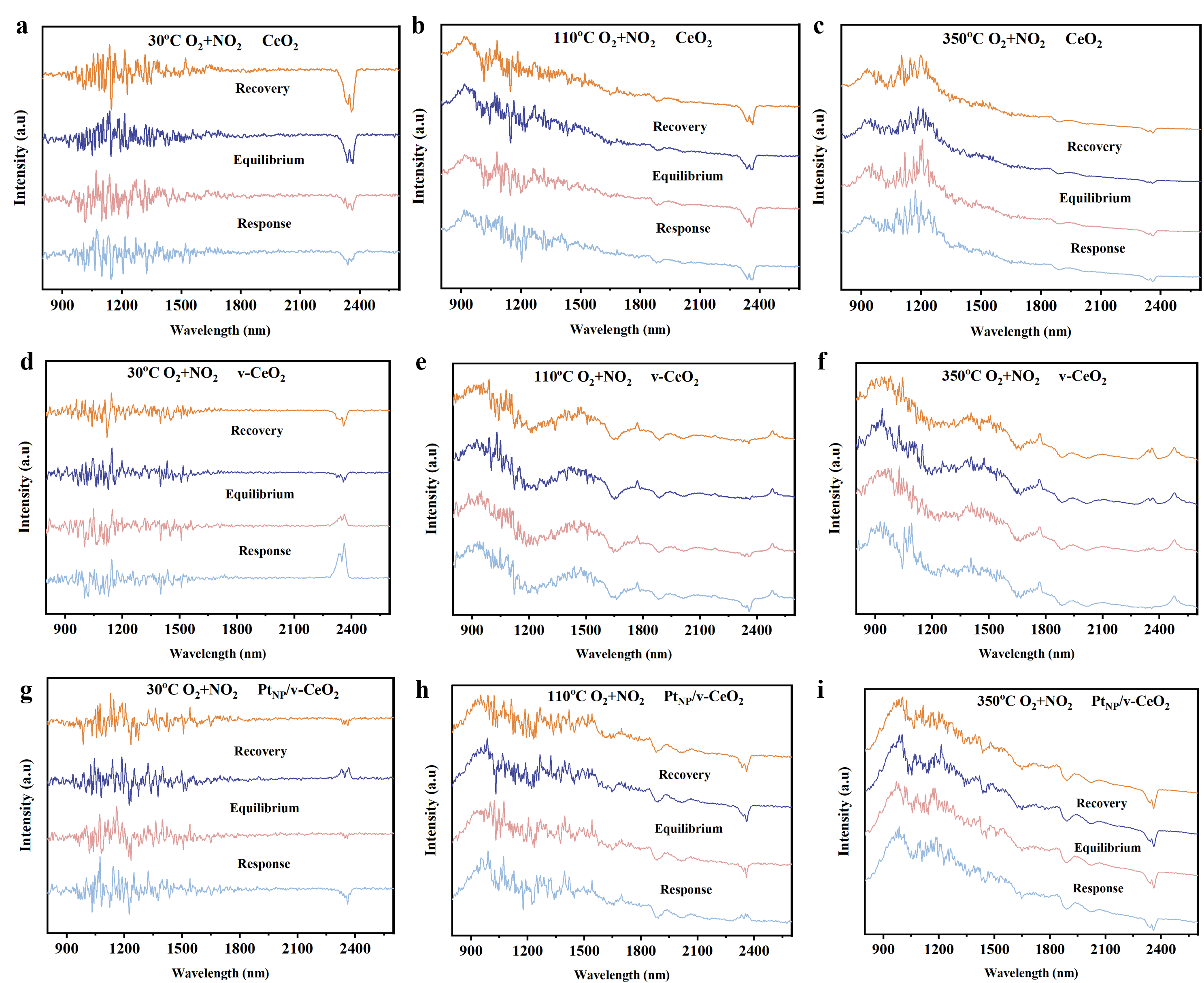


**Fig. S33** gas sensing reaction on the surface of (a-c) CeO_2_ and (d-f) v-CeO.


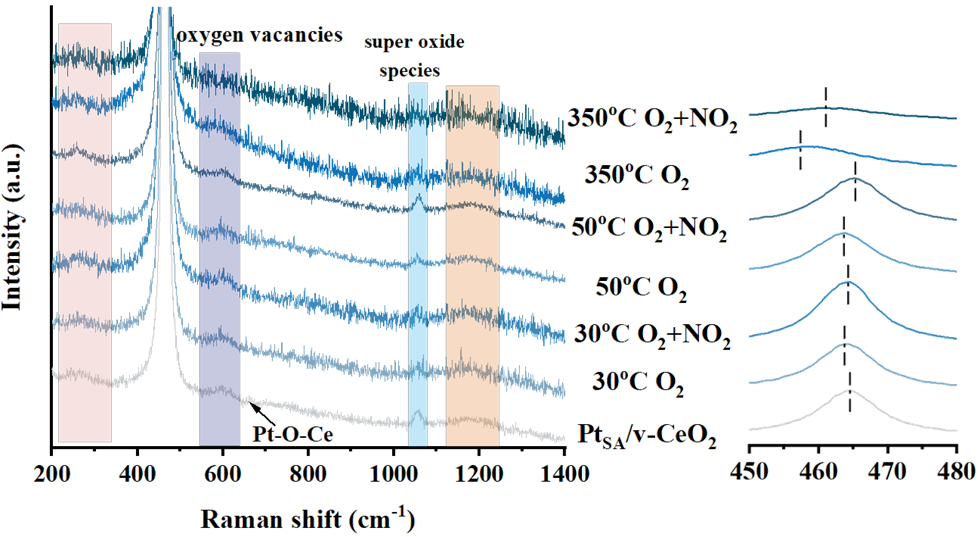


**Fig. S34** In-situ Raman analysis of Pt_SA_/v-CeO_2_ at different temperature under O_2_ and NO_2_.


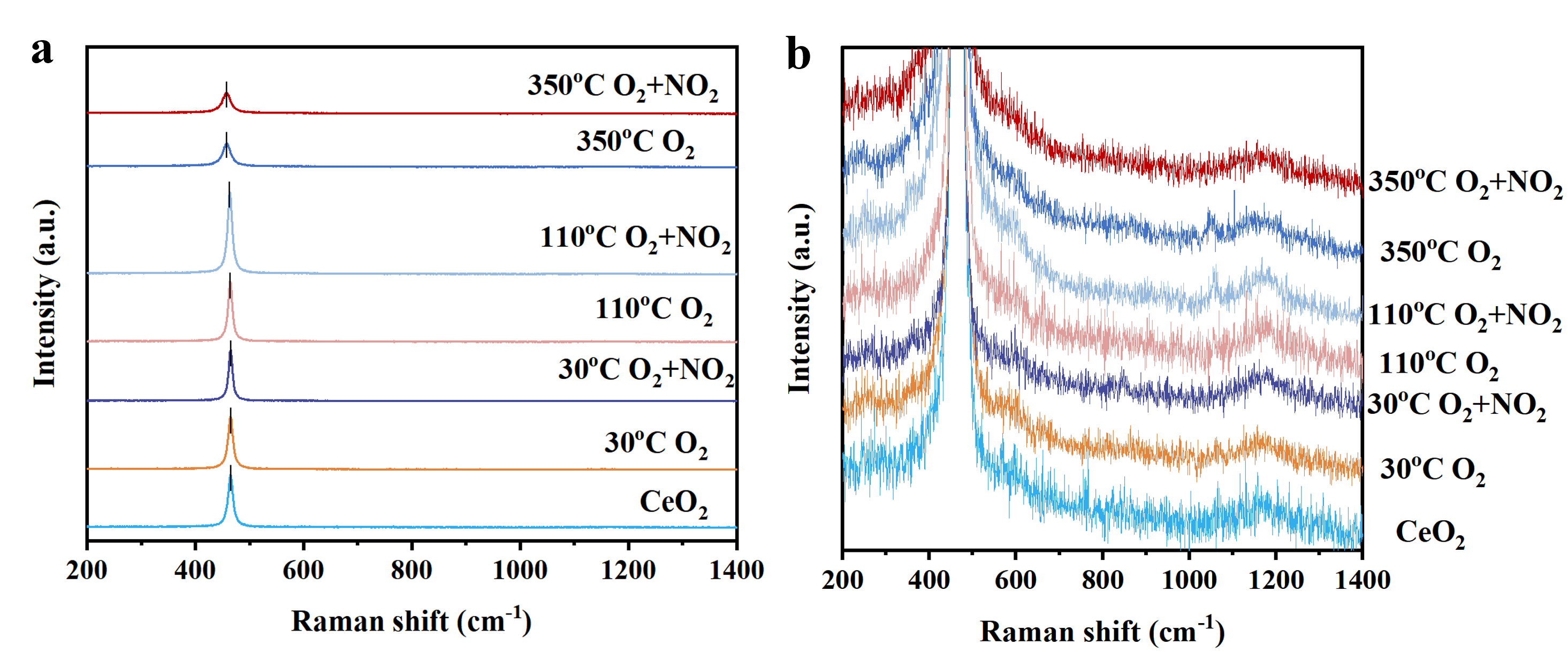


**Fig. S35** (a,b) In-situ Raman analysis of CeO_2_ at different temperature under O_2_ and NO_2_.

**III. Supplementary Tables**

Table S1. ICP analysis of Pt_SA_/v-CeO_2_.

|  | **m0(g)** | **mg/kg** | **W(%)** |
| --- | --- | --- | --- |
| 1 | 0.0456 | 3487.50 | 0.3488 |
| 2 | 0.0456 | 3559.43 | 0.3559 |
| 3 | 0.0456 | 3575.86 | 0.3576 |

Table S2. EXAFS fitting parameters at the Ce L-edge for various samples. CN^a^: coordination numbers; R^b^: bond distance; σ^2c^: Debye-Waller factors; ΔE_0_^d^: the inner potential correction. R factor: goodness of fit.

|  | shell | CN*^a^* | R*^b^*(Å) | σ^2^*^c^*(Å^2^) | ΔE_0_*^d^*(eV) | R factor |
| --- | --- | --- | --- | --- | --- | --- |
| CeO_2_ | Ce-O | 8 | 2.33±0.01 | 0.0062 | 7.3±0.7 | 0.0051 |
| 800^o^C-CeO_2_ | Ce-O | 7.5±0.5 | 2.33±0.01 | 0.0051 | 7.1±1.1 | 0.0080 |
| Pt_SA_-CeO_2_ | Ce-O | 8.1±0.5 | 2.33±0.01 | 0.0063 | 6.9±0.9 | 0.0047 |

Table S3. Multi-exponential decay function fitting results for TAS dynamics of v-CeO_2_.

| Temperature(^o^C) | τ_1_ (ps) | τ_2_ (ps) |
| --- | --- | --- |
| 30^o^C | 0.02 | 4.16 |
| 110^o^C | 3.88 | 1.54 |
| 200^o^C | 3.49 | 0.70 |
| 350^o^C | 0.58 | 6.74 |

Table S4. Multi-exponential decay function fitting results for TAS dynamics of CeO_2_.

| Temperature(^o^C) | τ_1_ (ps) | τ_2_ (ps) |
| --- | --- | --- |
| 30^o^C | 2.82 | 3.23 |
| 110^o^C | 2.27 | 2.41 |
| 200^o^C | 1.95 | 1.53 |
| 350^o^C | 1.64 | 1.27 |
